# Exploration of the Main Antibiofilm Substance of *Lactobacillus plantarum* ATCC 14917 and Its Effect against *Streptococcus mutans*

**DOI:** 10.3390/ijms24031986

**Published:** 2023-01-19

**Authors:** Jingheng Liang, Yan Zhou, Guihua Tang, Ruixue Wu, Huancai Lin

**Affiliations:** 1 Hospital of Stomatology, Sun Yat-sen University, Guangzhou 510055, China; 2 Guangdong Provincial Key Laboratory of Stomatology, Sun Yat-sen University, Guangzhou 510055, China; 3 School of Pharmaceutical Sciences, Sun Yat-sen University, Guangzhou 510006, China

**Keywords:** biofilm, lactic acid, *Streptococcus mutans*, dental caries, *Lactobacillus plantarum*

## Abstract

Dental plaque, a complex biofilm system established by cariogenic bacteria such as *Streptococcus mutans* (*S. mutans*), is the initiator of dental caries. Studies have found that the cell-free supernatant (CFS) of *Lactobacilli* could inhibit *S. mutans* biofilm formation. However, the main antibiofilm substance of the *Lactobacilli* CFS that acts against *S. mutans* is unclear. The present study found that the CFS of *Lactobacillus plantarum* (*L. plantarum*) ATCC 14917 had the strongest antibiofilm effect among the five tested oral *Lactobacilli*. Further bioassay-guided isolation was performed to identify the main antibiofilm substance. The antibiofilm effect of the end product, named **1-1-4-3**, was observed and the structure of it was elucidated by using Q-TOF MS, 2D NMR and HPLC. The results showed that several components in the CFS had an antibiofilm effect; however, the effect of **1-1-4-3** was the strongest, as it could reduce the generation of exopolysaccharides and make the biofilm looser and thinner. After structure elucidation and validation, **1-1-4-3** was identified as a mixture of lactic acid (LA) and valine. Additionally, LA was shown to be the main antibiofilm substance in **1-1-4-3**. In summary, this study found that the antibiofilm effect of the *L. plantarum* CFS against *S. mutans* was attributable to the comprehensive effect of multiple components, among which LA played a dominant role.

## 1. Introduction

Dental caries is a widely prevalent oral disease that seriously endangers human public health, with 2.5 billion people affected globally [1]. The initiator of dental caries is dental plaque, a complex biofilm system consisting of a collection of microbial communities enclosed by a matrix of exopolysaccharides (EPSs). The particular conditions of biofilm protect microbes from environmental damage factors, including host immune cells and drug treatments, and also impede the diffusion of acid, resulting in the demineralization of tooth enamel and, eventually, the occurrence of dental caries [2,3]. It has been reported that the pH of carious lesions could decrease down to a pH below 4.0, which is far below the pH threshold of enamel demineralization (5.5) [4,5].

*Lactobacilli* was the first caries-related microorganism discovered. The research on the relationship between *Lactobacilli* and caries can be traced back to the first half of the 20th century, when *Lactobacilli* was considered to be the main cariogenic bacteria [6]. Studies have found that the quantity of *Lactobacilli* in saliva and carious lesions is positively correlated to the decayed-missing-filled tooth index of permanent teeth [7]. Additionally, the detection rate of oral *Lactobacilli* in kindergarten children with caries was found to be significantly higher than that in caries-free children [8,9]. However, in recent decades, the role of *Lactobacilli* in dental caries has been doubted. It has been considered that *Lactobacilli* is not the initiating factor of caries, but rather an opportunistic invader of existing carious lesions due to their low pH milieu, which is beneficial to its survival [6]. It has also been reported that oral *Lactobacilli* can exert an antimicrobial effect against *Streptococcus mutans* (*S. mutans*), the main cariogenic bacteria, which has the ability to synthesize EPSs, acidogenicity and acid resistance. Studies have found that *Lactobacillus plantarum* (*L. plantarum*), *Lactobacillus casei* (*L. casei*), *Lactobacillus salivarius* (*L. salivarius*), *Lactobacillus gasseri* (*L. gasseri*) and *Lactobacillus fermentum* (*L. fermentum*) are all able to inhibit the biofilm formation of *S. mutans* [10,11,12]. As lactic acid bacteria (LAB), both *Lactobacilli* and *S. mutans* can produce lactic acid (LA) during sugar metabolism [13,14]. To figure out the role of *Lactobacilli* in dental caries, it is necessary to explore the mechanism of interaction between *Lactobacilli* and *S. mutans*.

Currently, the mechanism of inhibition of *Lactobacilli* against *S. mutans* is not yet clear. According to previous studies, the mechanism mainly includes the production of substances that can inhibit the growth of *S. mutans* [15,16,17], the coaggregation and competitive inhibition of adherence to the tooth surface [18,19], and the regulation of the expression of virulence-related genes in *S. mutans* [10,20]. A cell-free supernatant (CFS) of the culture broth of a microorganism is a liquid containing the metabolites generated during microbial growth and the residual nutrients of the medium used [14]. Studies found in the literature show that the CFS of *Lactobacilli* could exert an antimicrobial effect, and the antimicrobial compounds in the CFS mainly include LA, acetic acids, hydrogen peroxide, long-chain fatty acids and their esters, and proteinaceous compounds [14,21]. It was reported that the CFS of *Lactobacilli* contains biosurfactant-like substances that can inhibit the biofilm formation of *S. mutans* [22]. Additionally, the *Lactobacilli* CFS inhibited the growth and biofilm formation of *S. mutans* via bacteriocin-like substances [23]. Busarcevic et al. [24] extracted and purified a bacteriocin from the *L. salivarius* CFS, named LS1, that inhibited the growth of *S. mutans*.

Nevertheless, research about the inhibitive effect of the oral *Lactobacilli* CFS against *S. mutans* has not carried out with regard to the in-depth separation, purification or identification of a main antibiofilm component. In order to clarify how *S. mutans* and *Lactobacilli* interact with or compete against each other in carious lesions, and the distinct roles they contribute to dental caries, it is necessary to explore and determine the main antibiofilm substance against *S. mutans* generated by oral *Lactobacilli*. Therefore, this study aimed to extract and purify the main component in the CFS of oral *Lactobacilli* that can inhibit the biofilm formation of *S. mutans*, and to further clarify its properties and structures.

## 2. Results

### 2.1. Antibiofilm Effect of Cell-Free Supernatant (CFS) of Lactobacilli against Streptococcus mutans (S. mutans) 

**Figure 1:**
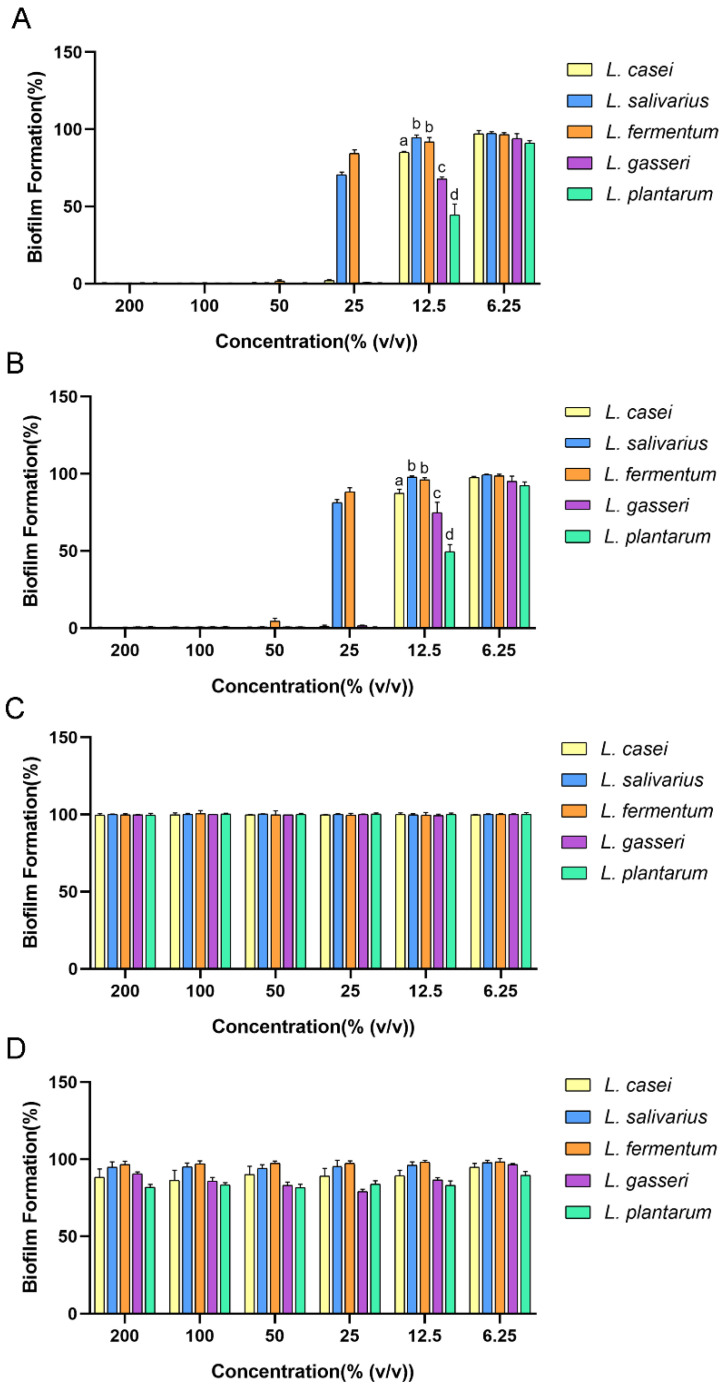
Antibiofilm effect of cell-free supernatant (CFS) of five *Lactobacilli* CFSs against *Streptococcus mutans* (*S. mutans*): (**A**) original CFS; (**B**) CFS treated with catalase; (**C**) CFS with pH adjusted to 6.5; (**D**) BHI with the same pH value as original CFS. The data are presented as the means ± SD. Different superscript letters for different values denote statistically significant differences (*p* ≤ 0.05).

In this study, *Lactobacilli* in the late logarithmic growth phase (Figure S1) was incubated for acquirement of CFS. As shown in Figure 1A, all original CFSs could inhibit 98% of biofilm formation of *S. mutans* at a concentration of over 50% (*v*/*v*). When the CFSs were diluted to the concentration of 12.5%, they performed different antibiofilm activities, among which the effect of the *L. plantarum* CFS was the strongest with a biofilm formation of only 44.4% (*p* ≤ 0.05).

As shown in the literature, the antimicrobial compounds in the CFS of *Lactobacilli* include organic acid and hydrogen peroxide. To preliminarily examine the properties of the main antimicrobial substance, the CFS was treated with catalase, and its pH was neutralized. After being treated with catalase, there was little change in the antibiofilm efficiency of the tested CFS (Figure 1B). Only the CFS of *L. salivarius* showed a reduced antibiofilm effect at the concentration of 25% after being treated with catalase (Figure S2). These results indicated that hydrogen peroxide was not the main antibiofilm substance in the CFS of tested *Lactobacilli*. In addition, this study found that the pH of the tested CFS ranged from 3.7 to 4.1. After the pH was adjusted to 6.5, all tested CFSs lost their antibiofilm ability (Figure 1C). In order to figure out whether the antibiofilm effect was attributed to the low-pH environment or to some substance that needed to function under acidic conditions, we examined the antibiofilm effect of a brain heart infusion (BHI) medium with the same pH as the corresponding original CFS (Figure 1D). The results showed that all tested BHIs had a certain antibiofilm effect; however, the ratios of biofilm formation were over 80% in all groups. These results indicated that the main antibiofilm component might be either a weak acid or a pH-dependent bioactive substance. As the CFS of *L. plantarum* showed the strongest antibiofilm activity among the tested CFSs, it was selected to be studied further to determine the main antibiofilm substance within it.

### 2.2. Isolation of the Main Antibiofilm Component of Lactobacillus plantarum (L. plantarum) CFS

**Figure 2:**
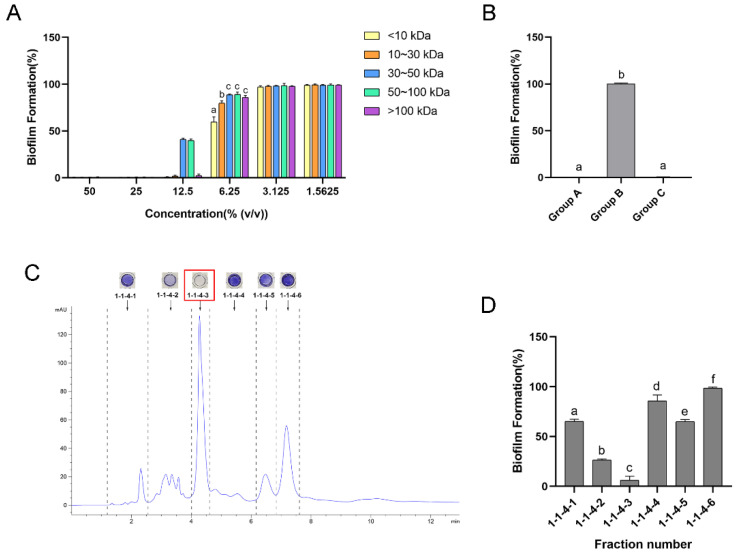
Isolation of the main antibiofilm component of *Lactobacillus plantarum* (*L. plantarum*) CFS. (**A**) Antibiofilm effect of fractions of *L. plantarum* CFS with different molecular weights. (**B**) The effect of pH on the antibiofilm ability of the fraction weighing less than 10 kDa of *L. plantarum* CFS. Group A: the fraction weighing less than 10 kDa. Group B: the fraction weighing less than 10 kDa with pH adjusted to 6.5. Group C: the fraction weighing less than 10 kDa with pH adjusted to 6.5 and then returned to the original value. (**C**) High-performance liquid chromatography (HPLC) chromatogram of Fr. **1-1-4**. Arrows indicate the peak fractions, with the results of crystal violet staining above. (**D**) Antibiofilm effect of the six isolated fractions (Frs. **1-1-4-1**~**1-1-4-6**). The data are presented as the means ± SD. Different superscript letters for different values denote statistically significant differences (*p* ≤ 0.05).

After ultrafiltration, the CFS of *L. plantarum* was divided into five fractions with different molecular weights, including less than 10 kDa, 10~30 kDa, 30~50 kDa, 50~100 kDa and greater than 100 kDa. The antibiofilm ability of each fraction is shown in Figure 2A. The results illustrate that all fractions could inhibit over 99.5% of biofilm formation at the concentration of 25%. When the fractions were diluted to the concentration of 6.25%, the difference in the inhibitive effect appeared. As shown in the results, the fraction with a molecular weight of less than 10 kDa showed the strongest effect, with a biofilm formation of 59.7% (*p* ≤ 0.05). Therefore, the fraction weighing less than 10 kDa was selected for further separation.

As the pH environment of the eluent cannot be well controlled in the subsequent separation, to further study the influence of pH, we tested the antibiofilm ability of the fraction weighing less than 10 kDa with different treatments. As shown in Figure 2B, in Group A, the fraction weighing less than 10 kDa in the CFS can inhibit 99.7% of biofilm formation at its original pH value (pH = 3.7). However, it lost its inhibitive effect when the pH was adjusted to 6.5 (Group B). Interestingly, when the pH was returned to the original value (Group C), the antibiofilm effect also recovered, with a 99.6% inhibition of biofilm formation, which was not significantly different from Group A (*p* > 0.05). These results indicate that the influence of pH on the antibiofilm ability of the active substance was reversible. 

During the further separation, the antibiofilm activity of each fraction was determined. The high-performance liquid chromatography (HPLC) chromatogram and antibiofilm effect of each fraction that was examined before the final separation are shown in Figures S3–S6. The fractions with the strongest effect were further separated. Fr. **1-1-4-3** was finally isolated as the main antibiofilm component, as it had the strongest antibiofilm effect with a narrow and symmetrical peak shape, as shown in the final separation (Figure 2C,D).

### 2.3. Antibiofilm Effect of Component ***1-1-4-3***

**Figure 3:**
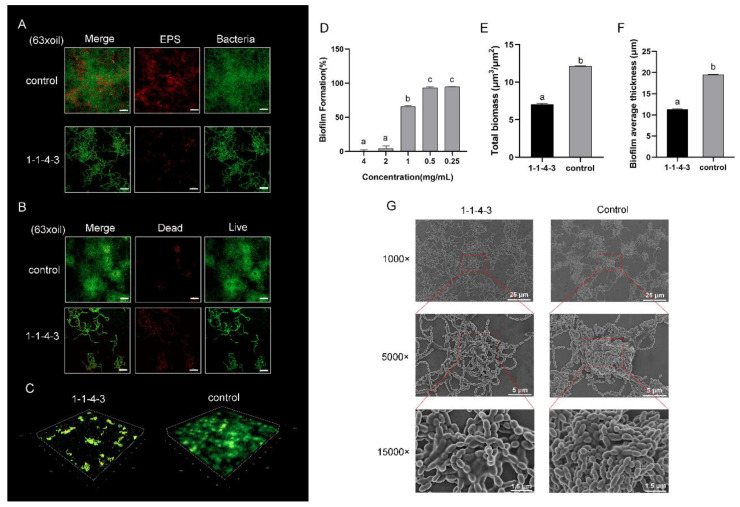
Antibiofilm effect of component **1-1-4-3**. (**A**–**C**) Confocal laser scanning microscopy (CLSM) images of biofilm of *S. mutans* in the absence and presence of **1-1-4-3**: (**A**) Green signal indicates microorganisms, whereas red signal indicates exopolysaccharides (EPSs). (**B**) Green signal represents live bacteria and red represents dead bacteria; (**C**) 3D structure of the biofilm. Scale bar: 10 µm. (**D**) Effect of dose on the antibiofilm ability of **1-1-4-3**. (**E**,**F**) Total biomass and biofilm average thickness of *S. mutans* biofilm in **1-1-4-3** and control group. (**G**) Scanning electron microscopy (SEM) images of biofilm of *S. mutans* in the absence and presence of **1-1-4-3**. Microstructure was observed and red boxes at low magnification were enlarged to a higher magnification. Different superscript letters for different values denote statistically significant differences (*p* ≤ 0.05).

The results showed that the antibiofilm effect of **1-1-4-3** was dose-dependent, with a biofilm formation of 4.2% and 65.9% at a concentration of 2 mg/mL and 1 mg/mL, respectively (Figure 3D). To further study the impact of **1-1-4-3** on the biofilm formation of *S. mutans*, we observed the biofilm in the presence or absence of **1-1-4-3** with confocal laser scanning microscopy (CLSM) and scanning electron microscopy (SEM). In Figure 3A, the green signal represents bacteria and the red signal represents the distribution of EPSs. It is obvious that after being treated with **1-1-4-3**, the EPSs in the biofilm reduced significantly, showing a loose connection among the bacteria. Furthermore, in Figure 3B, the green signal represents live bacteria and the red represents dead bacteria. It was found that after being treated with **1-1-4-3**, the total amount of bacteria decreased and the biofilm became obviously looser than that in the control group. Clumps of biofilm decreased and the biofilm became thinner, with weaker connections among bacterium and an apparent reduction in the total biofilm biomass (*p* ≤ 0.05) (Figure 3C,E). The average thickness of the biofilm exposed to **1-1-4-3** was 11.3 μm, whereas the control was 19.6 μm (*p* ≤ 0.05) (Figure 3F). In the images captured by SEM (Figure 3G), it can be seen that the biofilm treated with **1-1-4-3** became flat, and the clumps of biofilm were loose and weak. 

### 2.4. Characterization and Validation of Component ***1-1-4-3***

#### 2.4.1. ^1^H and ^13^C Nuclear Magnetic Resonance (NMR) and Quadrupole Time-of-Flight Mass Spectrometry (Q-TOF-MS) Data for Component **1-1-4-3**

^1^H NMR and ^13^C NMR data for **1-1-4-3** are shown in Table 1. Positive HR-ESI-MS showed a signal at *m*/*z* 118.0863 (calcd. for C_5_H_12_NO_2_^+^ [M + H]^+^ 118.0863) and negative HR-ESI-MS showed a signal at *m*/*z* 89.0244 (calcd. for C_3_H_5_O_3_^−^ [M – H]^−^ 89.0244) (Figures S7 and S8).

#### 2.4.2. Analysis of NMR and Q-TOF-MS Data

**Figure 4:**
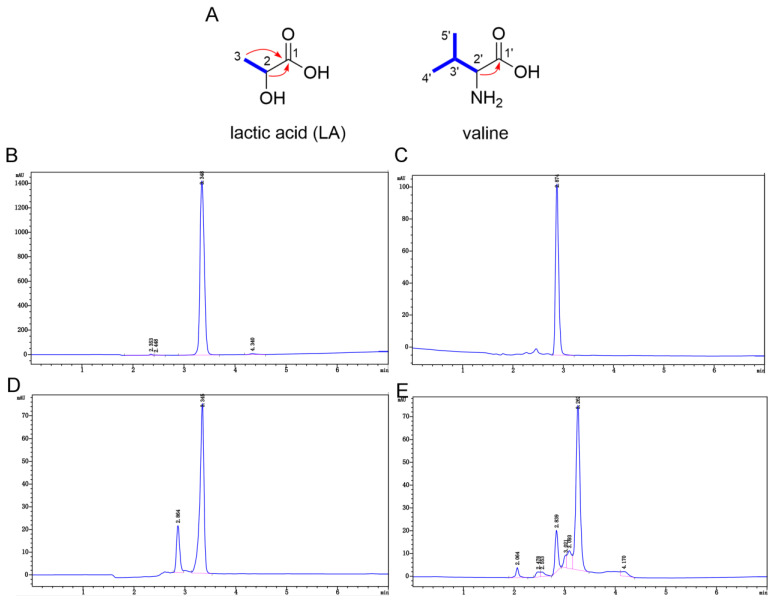
Characterization and validation of component **1-1-4-3.** (**A**) The^1^H–^1^H COSY (blue bold) and key HMBC (red arrow) correlations of **1-1-4-3**. (**B**~**E**) HPLC spectrum of lactic acid (LA) (**B**), valine (**C**), mixture of LA and valine (**D**), and **1-1-4-3** (**E**).

The ^1^H NMR and ^13^C NMR spectra of **1-1-4-3** showed two groups of resonance signals with a ratio of 2:1, which indicated that **1-1-4-3** was a mixture (Figures S9 and S10). Next, the two groups of high and low signals were analyzed. One secondary methyl (*δ*_H_ 1.22, d, (*J* = 6.9 Hz); *δ*_C_ 21.0 (CH_3_)), an oxygenated methine (*δ*_H_ 4.02, q, (*J* = 6.9 Hz); *δ*_C_ 66.3 (CH)) and one carbonyl (*δ*_C_ 176.9 (C)) were distinguished in the group with high signals in the ^1^H NMR, ^13^C NMR and DEPT spectra (Figures S11 and S12). The ^1^H–^1^H COSY correlation between the protons at *δ*_H_ 1.22 (H-3) and the proton at *δ*_H_ 4.02 (H-2) (Figure S13), as well as the HMBC correlations between H-3 and H-2 and the carbonyl (*δ*_C_ 176.9, C-1) (Figure S14), suggest that the secondary methyl, the oxygenated methine and the carbonyl were connected to form the structure of LA (Figure 4A). The quasimolecular ion peak at *m*/*z* 89.0244 (calcd. for C_3_H_5_O_3_^−^ [M − H]^−^ 89.0244) in the HR-ESI-MS further supported the presence of LA as a major constituent in **1-1-4-3**. As for the low-signal group, the ^1^H NMR, ^13^C NMR and DEPT spectra displayed characteristic signals for two secondary methyls (*δ*_H_ 0.89 and 0.93, each d, (*J* = 7.0 Hz); *δ*_C_ 18.1 and 19.3 (each CH_3_)); two methines, including a nitrogenated one (*δ*_H_ 3.12, d, (*J* = 3.6 Hz); *δ*_C_ 59.9 (CH)); and a carbonyl (*δ*_C_ 170.0 (C)). As shown in Figure 4A, the observed ^1^H–^1^H COSY correlations between H-2′/H-3′ and H-3′/H-4′(H-5′) indicated the moiety of CH-2′–CH-3′(–CH_3_-5′)–CH_3_-4′, which was linked to the carbonyl (*δ*_C_ 170.0, C-1′) by the key HMBC correlation between H-2′ and C-1′. Therefore, the component with low content was determined to be valine, whose molecular weight was also consistent with the HR-ESI-MS ion peak at *m*/*z* 118.0863 (calcd. for C_5_H_12_NO_2_^+^ [M + H]^+^ 118.0863). In addition, the 1D NMR and HPLC analysis of the authentic samples, LA and valine, as well as an analysis of their mixture (LA and valine at a ratio of 2:1), were also performed. By comparing the 1D NMR data (Table 1 and Figures S15–S22) and HPLC spectra (Figure 4B–E) of **1-1-4-3** with those of LA, valine, and their mixture, it was further confirmed that **1-1-4-3** contained LA and valine. 

### 2.5. Verification of the Role of Lactic Acid (LA) in the Antibiofilm Activity

#### 2.5.1. Antibiofilm Effect of Component **1-1-4-3**, LA (d/l-), Valine (d/l-) and the Mixture of LA and Valine

**Figure 5:**
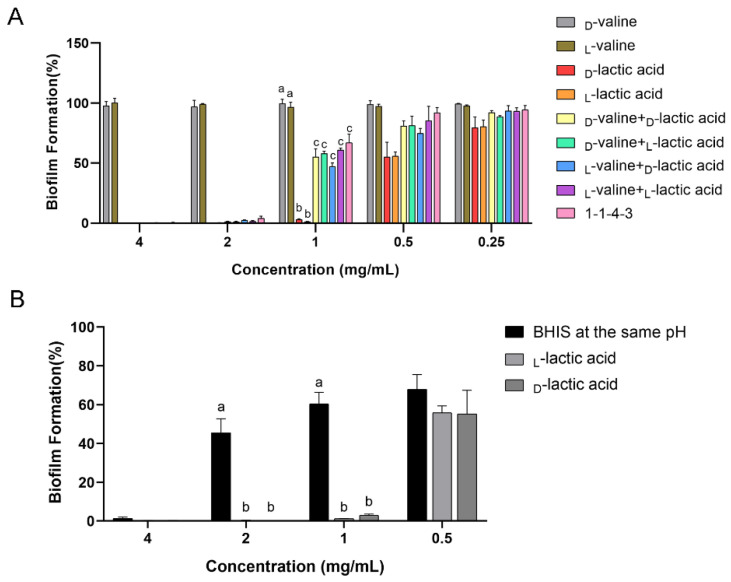
Verification of the role of LA in antibiofilm activity. (**A**) Antibiofilm effect of **1-1-4-3**, valine (d/l-), LA (d/l-) and the mixture of valine and LA. (**B**) Antibiofilm effect of LA and BHIS at the same pH as LA at each concentration. The data are presented as the means ± SD. Different superscript letters for different values denote statistically significant differences (*p* ≤ 0.05).

As for the antibiofilm effect (Figure 5A), LA (d/l-) showed a strong antibiofilm ability, with over 97% of biofilm formation inhibited at the concentration of 1 mg/mL. However, valine (d/l-) did not show an inhibitive effect on biofilm formation at all tested concentrations. The mixture of LA and valine showed a weaker antibiofilm effect compared with that of LA (d/l-), and its effect was similar to that of **1-1-4-3** (*p* > 0.05). These results indicate that LA was the antibiofilm component within **1-1-4-3**. Moreover, the effect between the two chiral LAs was not significantly different, and neither was the effect among the four kinds of mixtures (*p* > 0.05).

#### 2.5.2. The Antibiofilm Activity of LA and Low-pH Environment

In order to figure out the role of LA, we compared the antibiofilm activity of LA and of BHI broth with 1% (*w*/*v*) sucrose (1% BHIS) that was at the same pH as the LA at each concentration. The pH of LA at the concentrations of 0.5, 1, 2 and 4 mg/mL was 3.6, 3.5, 3.4 and 3.2, respectively. As shown in Figure 5B, the 1% BHIS with the same pH had a certain antibiofilm effect, which indicated that the low-pH environment did have an effect on the antibiofilm activity. However, d/l- LA at 1 and 2 mg/mL had a significantly stronger effect than that of the 1% BHIS with the same pH (*p* ≤ 0.05). The results indicate that in the CFS of *L. plantarum*, the low-pH environment played a certain role in the antibiofilm activity; however, LA also displayed strong antibiofilm activity in its undissociated form. In addition, to figure out the mechanism of inhibition of LA on the biofilm formation of *S. mutans*, we examined the minimal inhibitory concentration (MIC) of LA. The results showed that the MIC of LA (d/l-) was 1 mg/mL. Additionally, the minimum biofilm inhibitory concentration to inhibit 99% biofilm (MBIC_99_) of d/l- LA was 1.30 and 1.03 mg/mL. The MIC was close to the MBIC_99_, which indicates that the inhibition mechanism of the *L. plantarum* CFS on the biofilm formation of *S. mutans* may be mainly attributed to the inhibition of the growth of *S. mutans* by LA. 

## 3. Discussion

Previous studies have reported that *Lactobacilli* is dominant in dentine caries [25]. Additionally, the detection of oral *Lactobacilli* together with *S. mutans* has been correlated to the severity of dental caries [8]. However, as described above, the CFS of *Lactobacilli* was found to be inhibitive against *S. mutans*, the main cariogenic bacteria. The pathogenic mechanism of *Lactobacilli* in promoting caries and the interaction between *Lactobacilli* and *S. mutans* remain unclear.

The current study found that hydrogen peroxide played a small role in the antibiofilm activity of the five selected CFSs. The results were consistent with those of a previous study [10], which might indicate that the amount of hydrogen peroxide produced by the selected *Lactobacilli* was small, and, thus, hydrogen peroxide was not the main antibiofilm substance against *S. mutans* in the CFS. Since hydrogen peroxide is naturally unstable, further experiments can be carried out to quantify hydrogen peroxide in the five tested CFSs and clarify the role of hydrogen peroxide. This study found that the active components of the five tested CFSs needed to exert an inhibitive effect under an acidic condition. According to previous studies, organic acid in the CFS of *Lactobacilli* can obviously improve the effect of antibiofilm substances [14]. Interestingly, the influence of pH on the crude extract was reversible. These results indicate that the main active component might be a weak acid that causes an ionization equilibrium [26], or is a pH-dependent substance.

In oral microbiology, there is a lack of studies on the bioassay-guided isolation of active metabolites in regard to the interaction between bacteria. There are many methods for the extraction of active components in natural products, including extraction, sedimentation, dialysis, HPLC, etc. [27,28,29]. In the present study, the main antibiofilm component against *S. mutans* was screened from the CFS of *L. plantarum* by ultrafiltration, ethanol precipitation and HPLC. Ultrafiltration processes offer several advantages in comparison to the traditional conventional process: retention of characteristics and the structure of components in natural products, high selectivity, simplicity in continuous operations, integration, and scaling up [30]. In addition, this study combined the use of normal- and reversed-phase HPLC. The separation efficiency of HPLC under gradient conditions is best described by the peak capacity, which represents the maximum number of components that could be theoretically separated on a given liquid chromatography column within a gradient time [31]. The combination of normal- and reversed-phase HPLC produces a high peak capacity and better orthogonality, and can dramatically improve the separation power of chromatography, according to the mathematical model established by Giddings [32]. Moreover, extraction was used less in this experiment, which can reduce the inflammation, toxicity and environmental pollution of organic solvents [33].

During the separation, it was found that, except for the fraction weighing less than 10 kDa, the fractions exerted a certain antibiofilm effect. According to the literature, the antibiofilm components weighing more than 10 kDa in the *Lactobacilli* CFS mainly include proteinaceous compounds [14]. These results indicate that the antibiofilm substances in the CFS of *L. plantarum* might contain macromolecular proteinaceous compounds. In addition, during the separation via reversed-phase HPLC, some components with a late peak also had certain antibiofilm activity, and the polarity of such components was low. According to previous studies, the CFS might contain long-chain fatty acids [14,21]. Furthermore, it was found that the elution times of the most active component in the normal- and reversed-phase HPLC were close. Therefore, the active component had a certain solubility in both water and organic solvents. These results indicate that the active component might contain amphiphilic bacteriocins or biosurfactants and organic acids soluble in water and organic solvents.

In this study, the fractions with the strongest antibiofilm effect were further separated. Additionally, the antibiofilm effect of the end product, **1-1-4-3,** was examined via CLSM and SEM. The results indicate that **1-1-4-3** can reduce the EPS and clustered biofilm formation while causing the biofilm to be scattered and sparse. According to the literature, the *L. casei* CFS can reduce the number of EPSs and proportion of water-insoluble glucan in the biofilm of *S. mutans* and reduce the surface roughness of the biofilm [34]. Through the structural identification using 2D NMR and MS, as well as through the HPLC analysis and activity verification, it was found that **1-1-4-3** was a mixture of LA and valine with a ratio of 2:1, and the active component was LA. The problem of proton overlapping resonance in 1D NMR was overcome by 2D NMR, which is more conducive for conducting a structural analysis of complex compounds [35]. 

This study found that the effect of LA was obviously stronger than that of the other components, and the active components exerted their effects under an acidic environment. In addition, LA was the main organic acid in the *Lactobacilli* CFS [14]. These results indicate that LA plays a dominant role in the antibiofilm effect of the *L. plantarum* CFS. Chen et al. [36] found that the concentration of LA in the CFS of *L. plantarum* can reach 23.3~27 mg/mL, which might explain why, after a series of dilution, the tested CFS still had a strong antibiofilm activity against *S. mutans*. It was reported that *Lactobacilli* can produce both LA isomers (d- and l-) or the racemic blend) [14]. In this study, the antibiofilm effect of d/l- LA was not significantly different, which proved that both chiral LAs in the CFS had an antibiofilm activity effect. 

According to the literature, despite the fact that *S. mutans* can produce and resist acid, its resistance against acid is limited. The current study found that both LA and BHIS with the same pH as LA had an antibiofilm effect, but the effect of LA was significantly stronger. It has been reported that a low-pH environment can inhibit the growth of *S. mutans*, and that its virulence is related to biofilm formation [37]. Cells of *S. mutans* grew at a slow rate in a medium with a pH of 5.3 and lost the ability to perform glycolysis when the pH dropped to 3.45 [5]. Meanwhile, according to previous studies, LA is the main end product of *S. mutans* metabolism under carbon excess. Additionally, excess undissociated LA could inhibit the growth of *S. mutans* [38]. Excessive LA may change the osmotic stresses in *S. mutans* cells, which leads to the inhibition of the activity of lactate dehydrogenase, resulting in the impairment of the ability of *S. mutans* to metabolize exogenous carbohydrates [39,40]. It was considered that only undissociated LA causes inhibition, not the lactate anions, because only the protonated species can be transported across the cytoplasmic membrane [38]. The phenomenon of the stronger effect of LA compared with the BHIS with the same pH might be explained by the fact that an LA solution can carry more hydrogen ions in the undissociated form. Furthermore, the undissociated LA can cross the membrane, resulting in the decrease in internal pH values and the impairment of the carbohydrate metabolism. In view of the antimicrobial mechanism of LA reported previously, the authors thought that the inhibition mechanism of the *L. plantarum* CFS on the biofilm formation of *S. mutans* may be mainly attributed to its inhibition of the growth of *S. mutans* due to LA. 

Considering that the detection of *Lactobacilli* has been associated with caries severity [8] and *Lactobacilli* can reduce the quantity of *S. mutans* in the oral cavity [41], it is speculated that the activity of *L. plantarum* in carious lesions may be described as follows: when the concentration of LA is below the tolerance threshold of *S. mutans*, *L. plantarum* produces LA together with *S. mutans*, promoting the progression of caries, whereas when the concentration of LA is above the tolerance threshold of *S. mutans* in a low-pH environment, LA and other substances in the CFS produced by *L. plantarum* inhibit the growth and biofilm formation of *S. mutans*, competing for dominance in the carious lesions. 

The findings of this study suggest that the interaction between *Lactobacilli* and *S. mutans* might promote dental caries due to excess LA. Therefore, the administration of *Lactobacilli* supplements as probiotics may need to be weighed against the possibility that they contribute to dental caries. However, the tested *L. plantarum* CFS contained other antibiofilm components which are possibly pH sensitive. In further studies, such components could be further separated to develop caries prevention agents with pH sensitivity and low toxicity that maintain the balance of the microbiome in the neutral environment. 

In conclusion, this study found that the antibiofilm effect of the *L. plantarum* CFS against *S. mutans* was attributable to the comprehensive effect of multiple components; however, LA plays a dominant role in the antibiofilm activity. 

## 4. Materials and Methods

### 4.1. Bacterial Strains, Culture Conditions and Growth Curves Determination

Three *Lactobacilli* strains, *L. plantarum* ATCC 14917, *L. casei* ATCC 393 and *L. gasseri* ATCC 33323, and *S. mutans* UA 159 were acquired from Guangdong Microbial Culture Collection Center (GDMCC; Guangzhou, China). The other two *Lactobacilli* strains, *L. fermentum* ATCC 14931 and *L. salivarius* ATCC 11741, were acquired from China General Microbiological Culture Collection Center (CGMCC; Beijing, China). The *Lactobacilli* strains and *S. mutans* were cultured in de Man–Rogosa–Sharpe (MRS) broth and BHI broth, respectively, at 37 °C under microaerophilic conditions (6% O_2_, 3.6% H_2_, 3.6% CO_2_, 86.8% N_2_). Growth curves of *Lactobacilli* and *S. mutans* were determined by counting viable cells seeded on the corresponding agar medium every two hours.

### 4.2. Preparation of CFS

The CFS of each *Lactobacilli* was prepared according to the modified protocol of Lin [23]. Briefly, bacteria at the late logarithmic growth phase were adjusted to 1 × 10^7^ CFU/mL and then incubated microaerophilically at 37 °C for 48 h. Subsequently, the spent culture was centrifuged (5000× *g*, 15 min, 4 °C), which was followed by filtration through a 0.22 μm filter (Merck Millipore, Boston, MA, USA) to acquire the CFS. Considering that the concentration of the CFS in the natural niche of biofilm should be 100% (*v*/*v*) or possibly even higher due to local diffusion obstruction, in this study, the CFS was concentrated at a final concentration of 200% (*v*/*v*) by freeze-drying.

The prepared CFS was divided into three portions, as described previously [10]. One portion was the original concentrated CFS, and its pH was tested. The other two portions were treated to eliminate the effect of pH and hydrogen peroxide. The effect of pH was neutralized by adjusting the pH value of the CFS to 6.5 ± 0.1 with 1 M of NaOH. Additionally, the effect of hydrogen peroxide was eliminated by treating the CFS with 0.5 mg/mL of catalase (Solarbio, Beijing, China). Furthermore, to study the role of the low-pH environment, we prepared the BHI with the same pH as the original concentrated CFS, which was adjusted with 1 M of HCl. The treated and untreated CFSs were stored at −20 °C.

### 4.3. Antibiofilm Assay of CFS on S. mutans

A previously described, a crystal-violet-based microtiter plate assay was performed to determine the inhibitive effect of the CFS on the biofilm formation of *S. mutans* [42]. Briefly, *S. mutans*, at a final concentration of 1.0 × 10^6^ CFU/mL in 2% BHIS, was cultured in 96-well flat-bottom microplates with or without the addition of the prepared CFS or BHI at the same volume, ensuring that the final volume of each well was 200 μL. The final concentrations of the tested CFSs were in the range of 6.25% to 200% (*v*/*v*). After incubation at 37 °C for 24 h, the biofilm was washed twice with phosphate-buffered saline (PBS) and fixed with methanol for 15 min. Later, the wells were air-dried for 30 min and the fixed biofilm was then stained with 0.1% crystal violet solution for 15 min. After that, the excess dye was removed and the biofilm was rinsed with water. The crystal violet stains were solubilized in 96% ethanol, and were shaken vigorously for 1 h until completely dissolved. Lastly, the optical densities were measured at the wavelength of 595 nm using a microplate reader. *S. mutans* was treated with MRS broth as the negative control according to the same procedure as the CFS. Biofilm formation was expressed as a ratio of crystal-violet-stained biofilms relative to the negative control. 

### 4.4. Ultrafiltration of L. plantarum CFS and Further Investigation of the Influence of pH

The original CFS of *L. plantarum* was fractionated with Amicon^®^ Ultra-15 Centrifugal Filter Devices with different cutoffs (MWCO), including 10 K, 30 K, 50 K and 100 K (10,000 MWCO, 30,000 MWCO, 50,000 MWCO and 100,000 MWCO) (Merck KGaA, Darmstadt, Germany), according to the manufacturer’s recommendations. Briefly, 15 mL of the CFS was centrifuged at 4000× *g* at 4 °C to reach the volume of 150~300 μL. The sample held by the filter was collected, whereas the eluted sample was subjected to further centrifuging by a device with a lower cutoff. The effect of all collected fractions on the biofilm formation of *S. mutans* was determined by using a crystal violet staining assay. 

To further test the impact of pH on the antibiofilm ability of the fraction screened by ultrafiltration, three groups (A~C) were set for the assay. Group A: the fraction screened by ultrafiltration was untreated. Group B: the pH value of the fraction was adjusted to 6.5 with 1 M of NaOH. Group C: the pH value of the fraction was first adjusted to 6.5 with 1 M of NaOH, and then was adjusted back to the original value with 1 M of HCl. 

### 4.5. Bioassay-Guided Isolation of the Main Antibiofilm Component

The active fraction was further separated by HPLC in the following process. Reversed- and normal-phase HPLC were both performed on an Agilent 1260 System (Agilent Technologies Inc., Palo Alto, CA, USA). Solvent mixtures are reported as %*v*/*v*, unless otherwise stated. Each fraction obtained by HPLC was subjected to the antibiofilm bioassay, and the fraction with the strongest effect was further separated. 

Briefly, the fraction screened out with ultrafiltration was concentrated via freeze-drying. Then, the sample was chromatographed over a ZORBAX SB-C18 column (5 μm, 4.6 mm × 150 mm, Agilent Technologies Inc., Palo Alto, CA, USA) using the eluent of acetonitrile (MeCN) and H_2_O that was supplemented with 0.1% trifluoroacetic acid (TFA) to obtain 12 fractions (Frs. **1**~**12**). The procedure for the linear gradient of MeCN supplemented with 0.1% TFA was as follows: 0~5 min, 5%; 8~20 min, 10%; 23~33 min, 15%; 36~46 min, 20%; 49~59 min, 30%; 69~79 min, 100%; and 84 min, 5%. Fr. **1** with the strongest activity was then further subjected to an AccucoreTM HILIC column (2.6 μm, 2.1 mm × 150 mm, Thermo Fisher Scientific Inc., Waltham, MA, USA), where the same eluent described above was used to obtain 12 fractions (Frs. **1-1**~**1-12**), and the linear gradient of MeCN supplemented with 0.1% TFA was as follows: 0~30 min, 5~30%. Fr. **1-1** with activity was pooled and lyophilized, and was then dissolved in cold anhydrous ethanol and kept at −20 °C overnight to precipitate polysaccharides, according to previous studies with some modifications [43]. The results of the bioassay indicated that the supernatant of Fr. **1-1** showed a strong antibiofilm effect, whereas its precipitate had no antibiofilm ability. Then, the supernatant of Fr. **1-1** was lyophilized and further separated by using an Eclipse XDB-C18 column (5 μm, 4.6 mm × 250 mm, Agilent Technologies Inc., Palo Alto, CA, USA), and was eluted with 5% isopropanol in methanol absolute/ultrapure water (95:5) to obtain 11 fractions (Frs. **1-1-1**~**1-1-12**). Fr. **1-1-4** with activity was then purified by using a ZORBAX NH_2_ column (5 µm, 4.6 mm × 250 mm, Agilent Technologies Inc., Palo Alto, CA, USA) and eluted with ethanol/*n*-hexane (70:30) to obtain 6 fractions (Frs. **1-4-1**~**1-4-6**). The results showed that Fr. **1-1-4-3** had the strongest antibiofilm activity with a narrow and symmetrical peak shape; thus, it was selected for further study. 

### 4.6. Examination of Antibiofilm Effect of Component ***1**-**1**-**4**-**3***

The effect of the antibiofilm activity of **1-1-4-3** at different doses was tested by using the crystal violet staining assay described above. Meanwhile, the morphological changes of the *S. mutans* biofilm were observed with SEM and CLSM. Briefly, *S. mutans*, at a final concentration of 1.0 × 10^6^ CFU/mL in 1% BHIS, was cultured on sterile glass coverslips or confocal dishes with or without the addition of **1-1-4-3**. For SEM observation, after incubation on sterile glass coverslips for 24 h, the biofilm was then fixed with 2.5% glutaraldehyde for 6 h, followed by dehydration using graded ethanol (50~100%). Subsequently, the biofilm was successively treated with a solution of *t*-butanol/ethanol (1:1) and a solution of *t*-butanol (each step for 15 min), and was then freeze-dried. Samples were sputtered with gold and observed via SEM (Zeiss, Oberkochen, Germany). For the CLSM observation, after incubation on confocal dishes for 24 h, the biofilm was rinsed with PBS three times and was then stained with SYTO 9 and propidium iodide using the LIVE/DEAD BacLight Bacterial Viability Kit (L7012, Invitrogen, Carlsbad, CA, USA), according to the manufacturer’s instructions. After that, the LSM 980 with Airyscan 2 (Zeiss, Oberkochen, Germany) was applied to observe the biofilm and capture images, which were then analyzed by using COMSTAT [44]. In addition, the distribution of EPSs in biofilm was observed with the Alexa Fluor 647 Dextran conjugate (Invitrogen Corp., Carlsbad, CA, USA) and SYTO 9. Briefly, with or without the addition of **1-1-4-3**, *S. mutans* was seeded in confocal dishes containing the culture medium with 2.5 μM of the Alexa Fluor 647 Dextran conjugate. After incubating for 24 h, 2.5 μM SYTO-9 was applied to label *S. mutans* cells, as previously described. The distribution of EPSs in the biofilm was observed by using the LSM 980 with Airyscan 2, as described above.

### 4.7. Structure Elucidation of Component ***1-1-4-3***

#### 4.7.1. NMR and Q-TOF-MS Analysis of Component **1-1-4-3**

Next, 1D and 2D NMR spectra were recorded on Bruker AVANCE III HD 600 MHz (Bruker, Karlsruhe, Germany) spectrometers at 300 K with TMS as the internal standard. Chemical shifts are expressed in ppm, with reference to the residual solvent signals (DMSO-*d*_6_ with *δ*_H_ 2.50 and *δ*_C_ 39.6). As for the mass spectrometry (MS) analysis, the positive and negative electrospray ionization (ESI) MS data were measured with Q-TOF-MS (Bruker maXis impact II, Bruker Daltonics, Karlsruhe, Germany). The MS raw data were obtained in the format from the Bruker Compass Data Analysis Viewer version 4.1 (Bruker Daltonics, Karlsruhe, Germany). According to the analysis of the 2D NMR and ESI-MS data of **1-1-4-3** (see Section 2.4), it was found that **1-1-4-3** was a mixture of LA (C_3_H_6_O_3_) and valine (C_5_H_12_NO_2_) at a ratio of about 2:1.

#### 4.7.2. NMR Analysis of Valine and LA Standards and HPLC Analysis of Component **1-1-4-3** and Its Standards

To further confirm the above results, the authentic samples of valine, LA (Macklin Inc., Shanghai, China) and their mixture were subjected to a 1D NMR analysis, according to the processes described in Section 4.7.1. In addition, the HPLC analyses of **1-1-4-3**, LA, valine and the 2:1 mixture of LA and valine were performed on an Agilent 1260 System using an Eclipse XDB-C18 column with the mobile phase consisting of 95% eluent A (ultrapure water) and 5% eluent B (5% isopropanol in methanol). The flow rate was 1.0 mL/min and the elution peak was detected at 220 nm. 

### 4.8. Exploration of the Role of LA in the Antibiofilm Activity

#### 4.8.1. Comparison of the Antibiofilm Effect of Component **1-1-4-3**, LA (d/l-), Valine (d/l-) and the Mixture of LA and Valine 

**1-1-4-3**, LA (d/l-), valine (d/l-) and a molar concentration of the 2:1 mixture of LA and valine were dissolved in 1% BHIS at a pH of 3.70 ± 0.05 until a series of gradient concentrations was established (0.25~4 mg/mL), and their antibiofilm activity was tested by using the crystal violet staining assay. Bacteria exposed to 1% BHIS with the same pH value were set as the negative control.

#### 4.8.2. Examination of the Antibiofilm Activity of LA and Low pH

To simulate the pH environment of the *L. plantarum* CFS, LA was diluted in 1% BHIS at a pH of 3.70 ± 0.05 until a series of gradient concentrations was established (0.5~4 mg/mL); then, the pH value of LA at each concentration was tested. In order to figure out the role of low pH, we prepared 1% BHIS with the same pH as LA at each concentration, whose pH was adjusted by using 1 M of HCl. The antibiofilm activity of LA at different concentrations and 1% BHIS with the same pH as LA at each concentration were then tested via the crystal violet assay. Bacteria exposed to 1% BHIS at the pH of 3.70 ± 0.05 were set as the negative control. The MBIC_99_ of LA was determined by constructing a dose–response curve via nonlinear fitting and calculating the concentration of IC_99_ via the GraphPad Prism version 8.0.2 software.

#### 4.8.3. Examination of the MIC of LA

The MIC of LA was measured by using the standard broth microdilution method, following the recommendations of the Clinical and Laboratory Standards Institute (CLSI) with some modifications [45]. Briefly, bacteria were diluted to a final concentration of 1.0×10^6^ CFU/mL and treated with LA at a series of gradient concentrations (0.5~4 mg/mL). Nontreated bacteria with broth were set as the negative control. The experiments were performed in 96-well microtiter plates. The MIC was determined as the lowest concentration of drugs that inhibited bacterial growth via visual inspection after 24 h of incubation.

### 4.9. Statistical Analysis

All the experiments were performed in triplicate, and the data are presented as the mean ± standard deviation. Statistical analysis was carried out using the GraphPad Prism version 8.0.2 software via a one-way analysis of variance (ANOVA) or unpaired *t*-test. Statistical significance was defined as *p* ≤ 0.05.

## Figures and Tables

**Table 1:** NMR spectroscopic data of component **1-1-4-3**, LA + valine, LA, and valine in DMSO-*d*_6_ at 600 (^1^H) and 150 (^13^C) MHz.

Position	1-1-4-3		LA + Valine		LA		Valine	
	*δ*_C_, Type	*δ*_H_, Mult. (*J* in Hz)	*δ*_C_, Type	*δ*_H_, Mult. (*J* in Hz)	*δ*_C_, Type	*δ*_H_, Mult. (*J* in Hz)	*δ*_C_, Type	*δ*_H_, Mult. (*J* in Hz)
1	21.0, CH_3_	1.22, d (6.9)	20.9, CH_3_	1.23, d (6.9)	20.9, CH_3_	1.23, d (6.8)		
2	66.3, CH	4.02, q (6.8)	66.2, CH	4.03, q (6.8)	66.3, CH	4.04, q (6.8)		
3	176.9, C		176.8, C		176.8, C			
1′	18.1, CH_3_	0.89, d (7.0)	18.2, CH_3_	0.91, d (7.0)			18.0, CH_3_	0.87, d (7.0)
2′	29.5, CH	2.16–2.09, m	29.5, CH	2.17–2.10, m			29.5, CH	2.19–2.06, m
3′	19.3, CH_3_	0.93, d (7.0)	19.0, CH_3_	0.94, d (7.0)			19.5, CH_3_	0.91, d (7.0)
4′	59.9, CH	3.12, d (3.6)	59.4, CH	3.28, d (3.9)			60.2, CH	3.00, d (3.5)
5′	170.0, C		170.3, C				170.4, C	

## Data Availability

The data presented in this study are available on request from the corresponding author.

## Supplementary Materials

The following supporting information can be downloaded at: https://www.mdpi.com/article/10.3390/ijms24031986/s1.
