# Supplementary material for: Exploration of the Main Antibiofilm Substance of Lactobacillus plantarum ATCC 14917 and Its Effect against Streptococcus mutans
Source: Int J Mol Sci. 2023 Jan 19;24(3):1986. doi: 10.3390/ijms24031986 (PMC9916977; doi:10.3390/ijms24031986)
Supplement: Supplementary file 1 [file ijms-24-01986-s001.zip › IJMS-Supplementary Materials-AA.pdf]

Supplementary Material

Table of Contents

Figure S1. Growth curves of *S. mutans* and five selected *Lactobacilli*

Figure S2. Biofilm formation of *S. mutans* in the presence of untreated and catalase-treated CFS of *L. salivarius*

Figure S3. Antibiofilm effect of fractions isolated by the ZORBAX SB-C18 column

Figure S4. Antibiofilm effect of fractions isolated by the Accucore™ HILIC column

Figure S5. Antibiofilm activity of the supernatant and precipitate in the assay of ethanol precipitation

Figure S6. Antibiofilm effect of fractions isolated by the Eclipse XDB-C18 column

Figure S7. Positive ESI-TOF spectrum of **1-1-4-3**

Figure S8. Negative ESI-TOF spectrum of **1-1-4-3**

Figure S9. ^{1}H NMR spectrum of **1-1-4-3** in DMSO-d_6

Figure S10. ^{13}C NMR spectrum of **1-1-4-3** in DMSO-d_6

Figure S11. DEPT spectrum of **1-1-4-3** in DMSO-d_6

Figure S12. HSQC spectrum of **1-1-4-3** in DMSO-d_6

Figure S13. ^{1}H-^{1}H COSY spectrum of **1-1-4-3** in DMSO-d_6

Figure S14. HMBC Spectrum of **1-1-4-3** in DMSO-d_6

Figure S15. ^{1}H NMR spectrum of LA in DMSO-d_6

Figure S16. ^{13}C NMR spectrum of LA in DMSO-d_6

Figure S17. DEPT spectrum of LA in DMSO-d_6

Figure S18. ^{1}H NMR spectrum of valine in DMSO-d_6

Figure S19. ^{13}C NMR spectrum of valine in DMSO-d_6

Figure S20. ^{1}H NMR spectrum of mixture of valine and LA in DMSO-d_6

Figure S21. ^{13}C NMR spectrum of mixture of valine and LA in DMSO-d_6

Figure S22. DEPT spectrum of mixture of valine and LA in DMSO-d_6

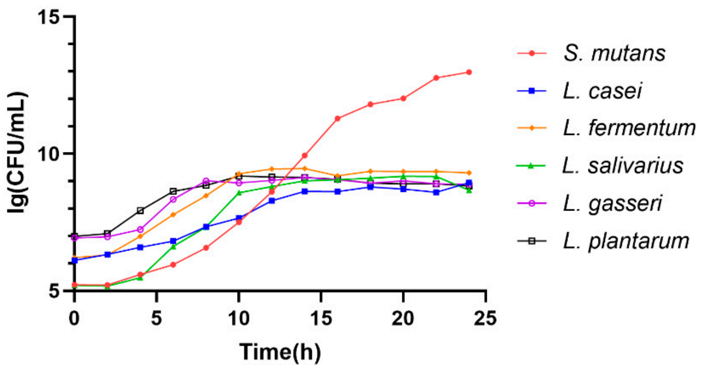

Figure S1. Growth curves of *S. mutans* and five selected *Lactobacilli*.

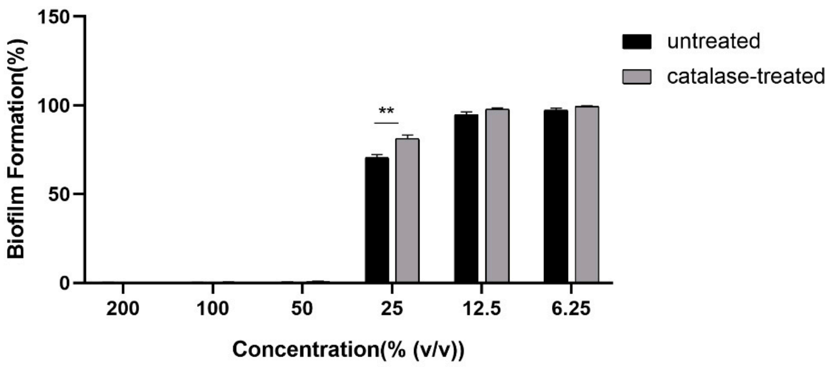

Figure S2. Biofilm formation of *S. mutans* in the presence of untreated and catalase-treated CFS of *L. salivarius*. The data are presented as the means ± SD. $^{**}p < 0.01$.

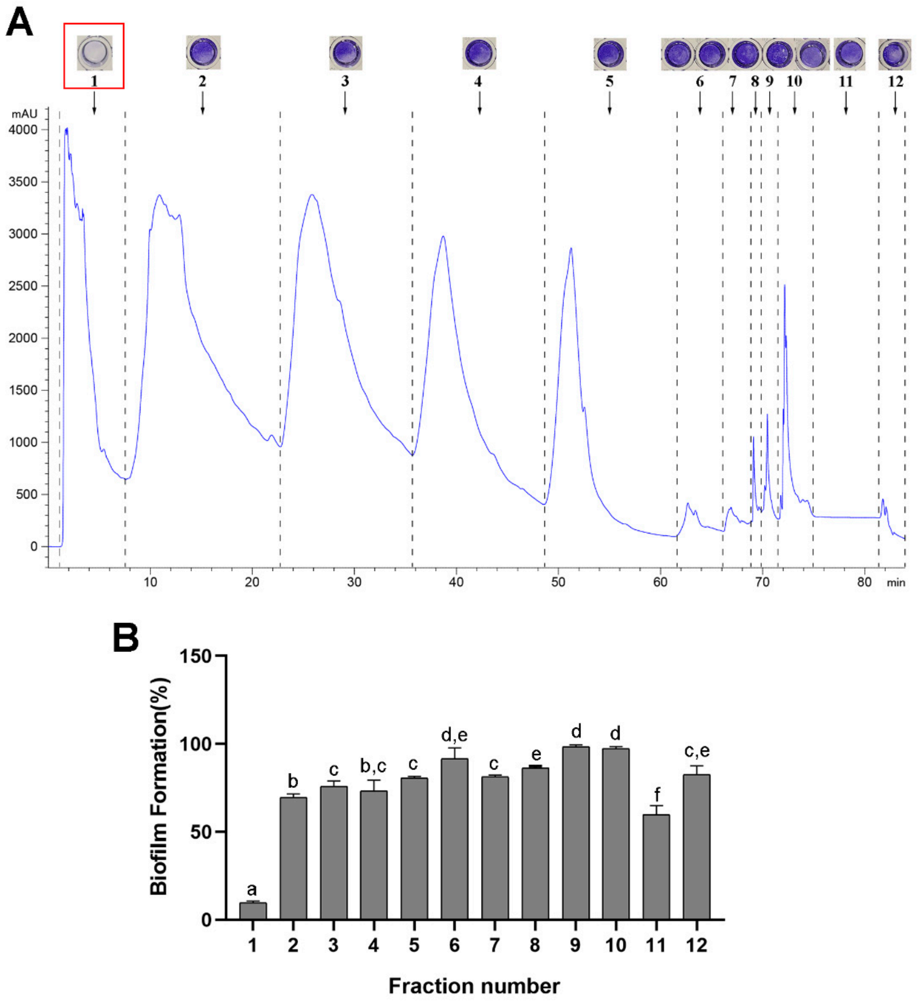

Figure S3. Antibiofilm effect of fractions isolated by the ZORBAX SB-C18 column. (A) HPLC chromatogram of the fraction weighing less than 10 kDa. Arrows indicate the peak fractions, with the results of crystal violet staining above. (B) Antibiofilm effect of the 12 isolated fractions (Frs. **1**~**12**). The data are presented as the means ± SD. Different superscript letters for different values denote significant statistically significant differences ($p \leq 0.05$).

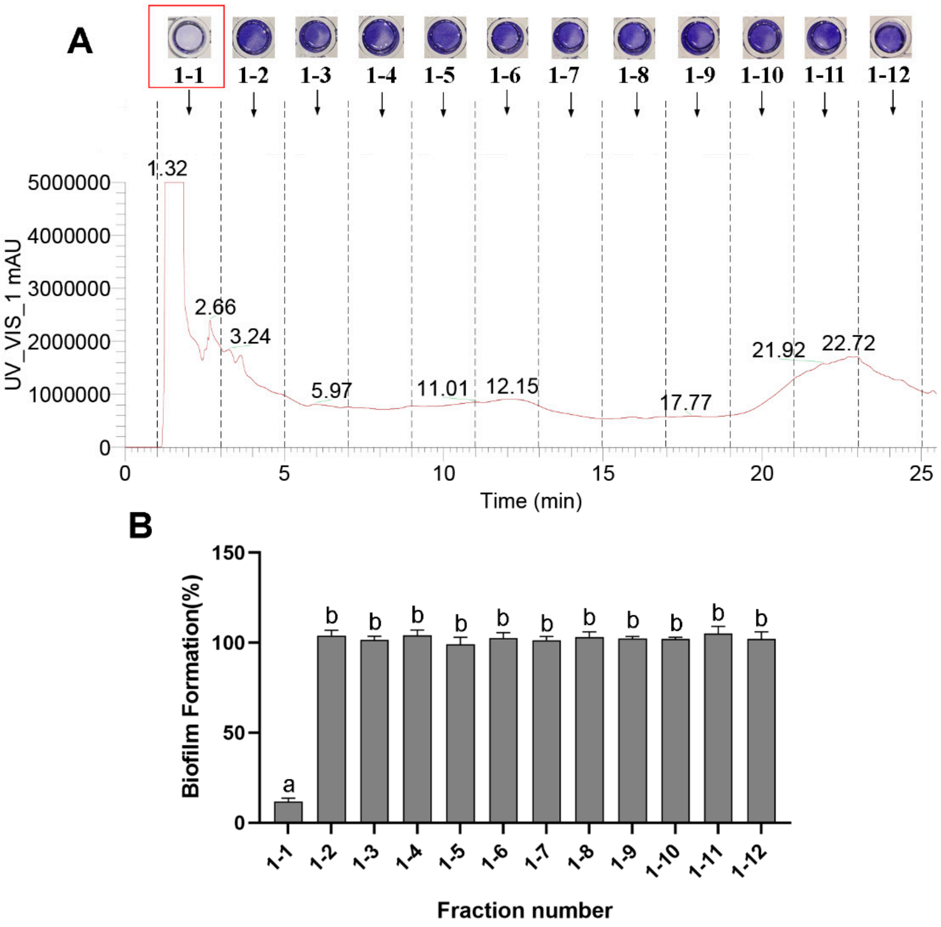

Figure S4. Anti-biofilm effect of fractions isolated by the Accucore™ HILIC column. (A) HPLC chromatogram of Fr. **1** isolated by the ZORBAX SB-C18 column. Arrows indicate the collected fractions, with the results of crystal violet staining above. (B) Antibiofilm effect of the 12 collected fractions (Frs. **1-1** ~ **1-12**). The data are presented as the means ± SD. Different superscript letters for different values denote significant statistically significant differences ($p \leq 0.05$).

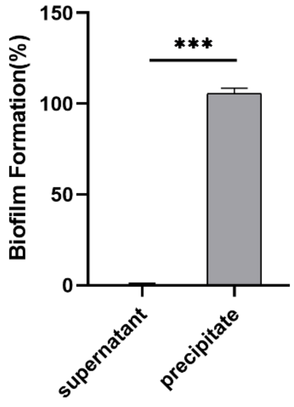

Figure S5. Anti-biofilm activity of the supernatant and precipitate of Fr. **1-1** in the assay of ethanol precipitation. The data are presented as the means ± SD. ***p < 0.001.

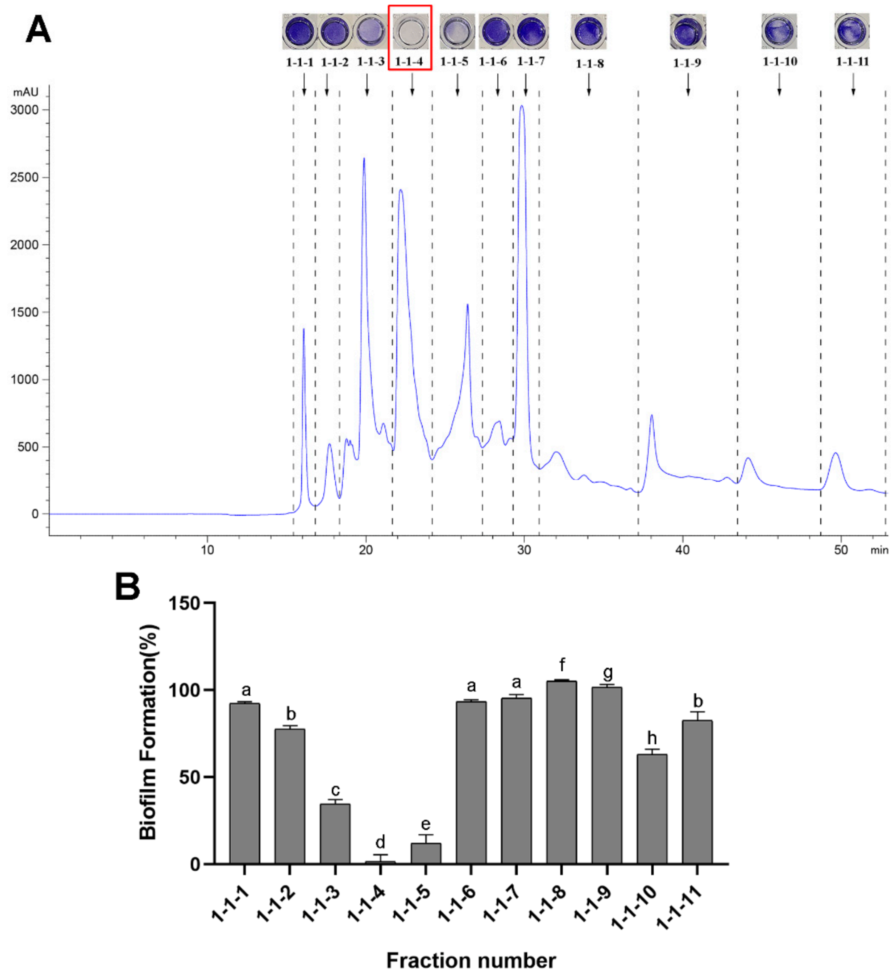

Figure S6. Antibiofilm effect of fractions isolated by the Eclipse XDB-C18 column. (A) HPLC chromatogram of the supernatant in ethanol precipitation assay. Arrows indicate the peak fractions, with the results of crystal violet staining above. (B) Antibiofilm effect of the 11 isolated fractions (Frs. **1-1-1** ~ **1-1-11**). The data are presented as the means ± SD. Different superscript letters for different values denote significant statistically significant differences ($p \leq 0.05$).

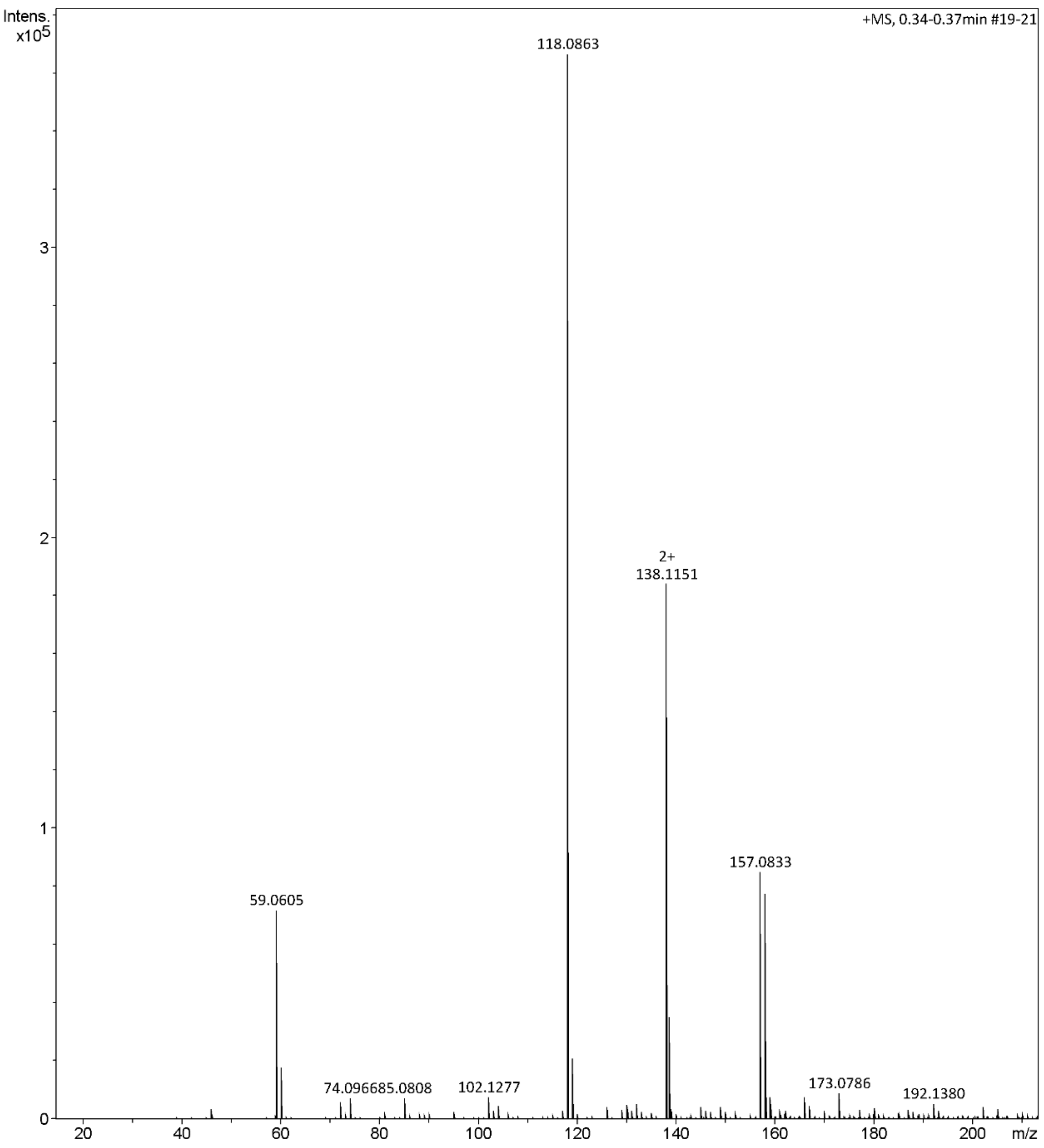

Figure S7. Positive ESI-TOF spectrum of **1-1-4-3**

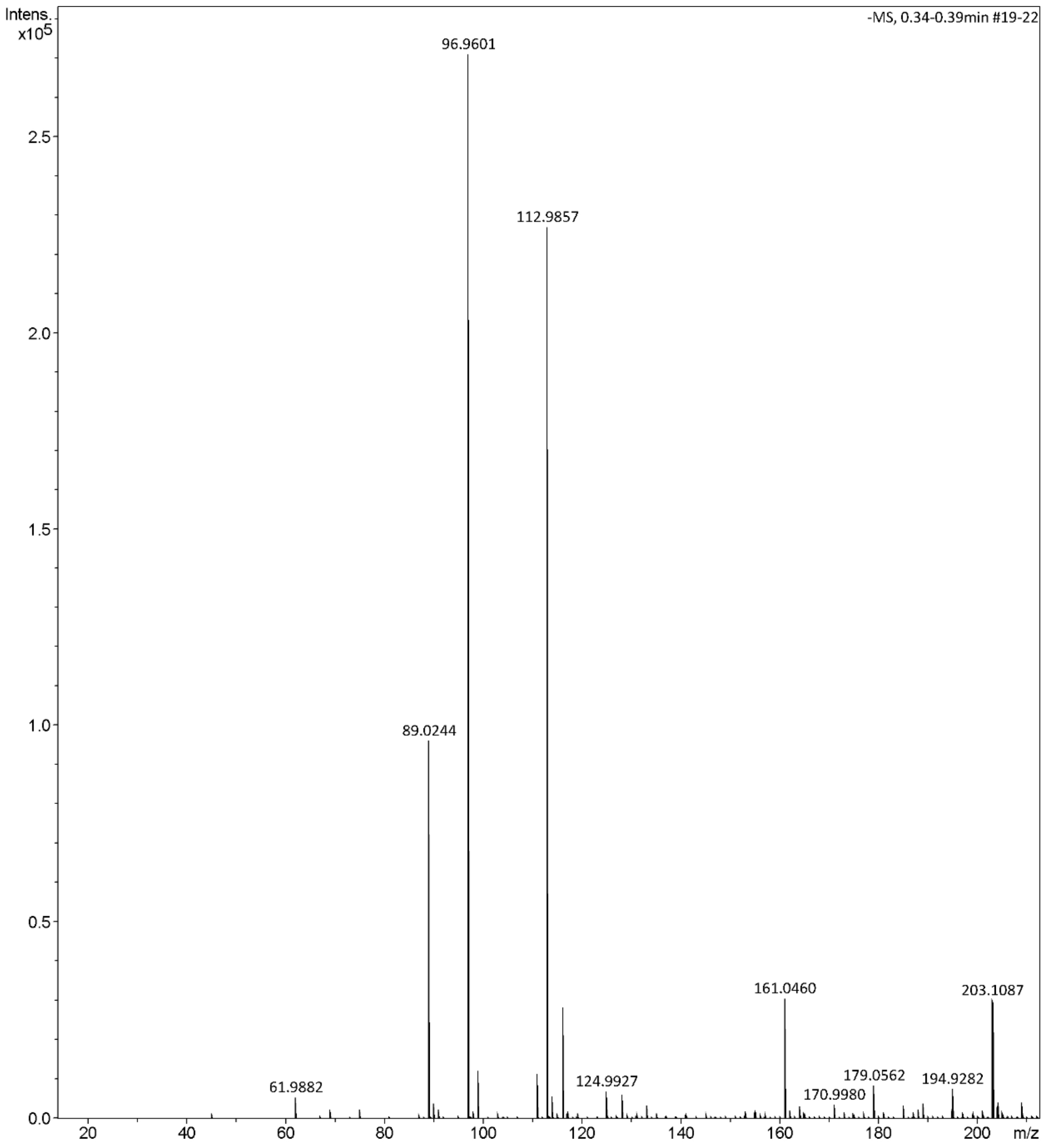

Figure S8. Negative ESI-TOF spectrum of **1-1-4-3**

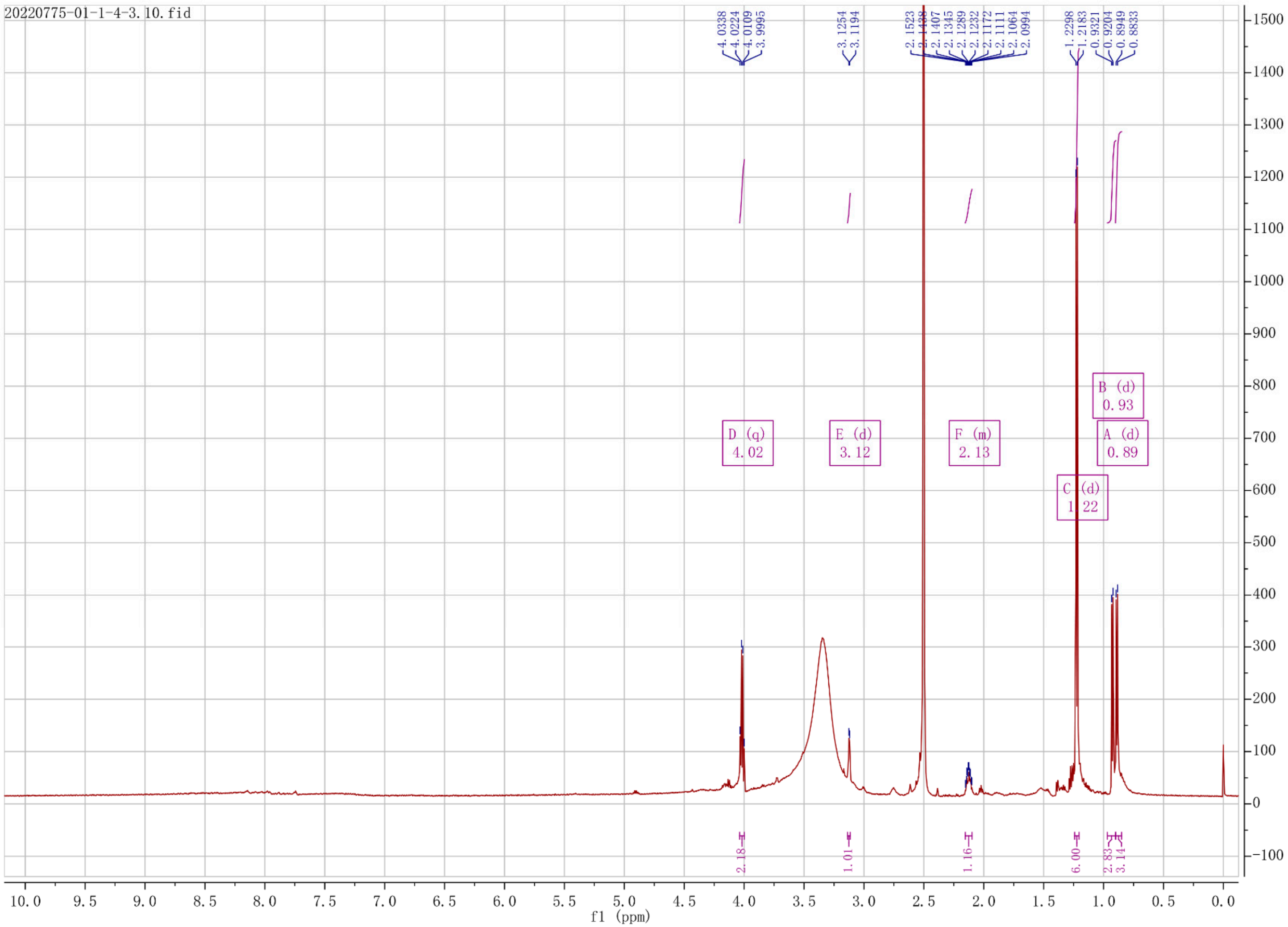

Figure S9. ^{1}H NMR spectrum of **1-1-4-3** in DMSO-d_6

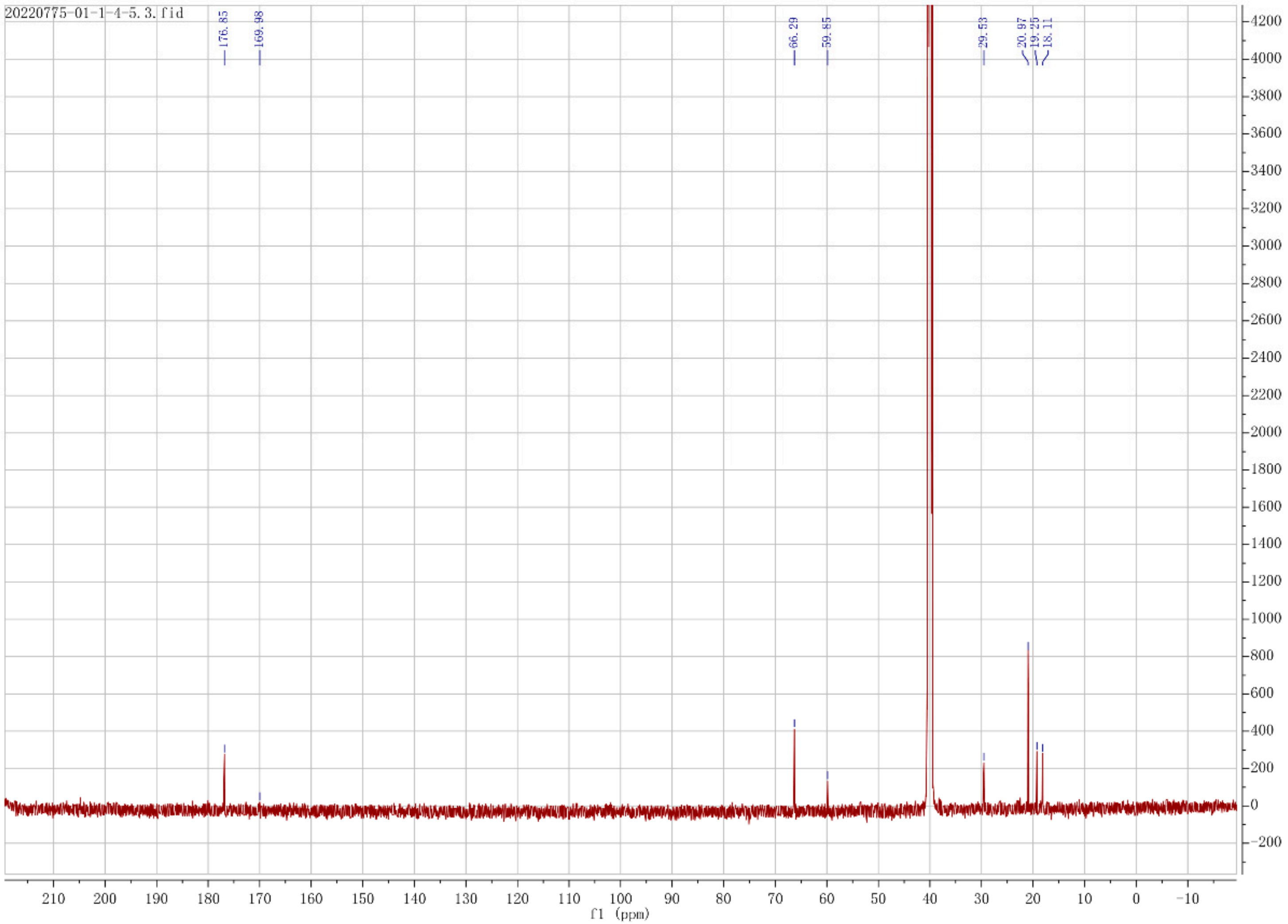

Figure S10. ^{13}C NMR spectrum of **1-1-4-3** in DMSO-d_6

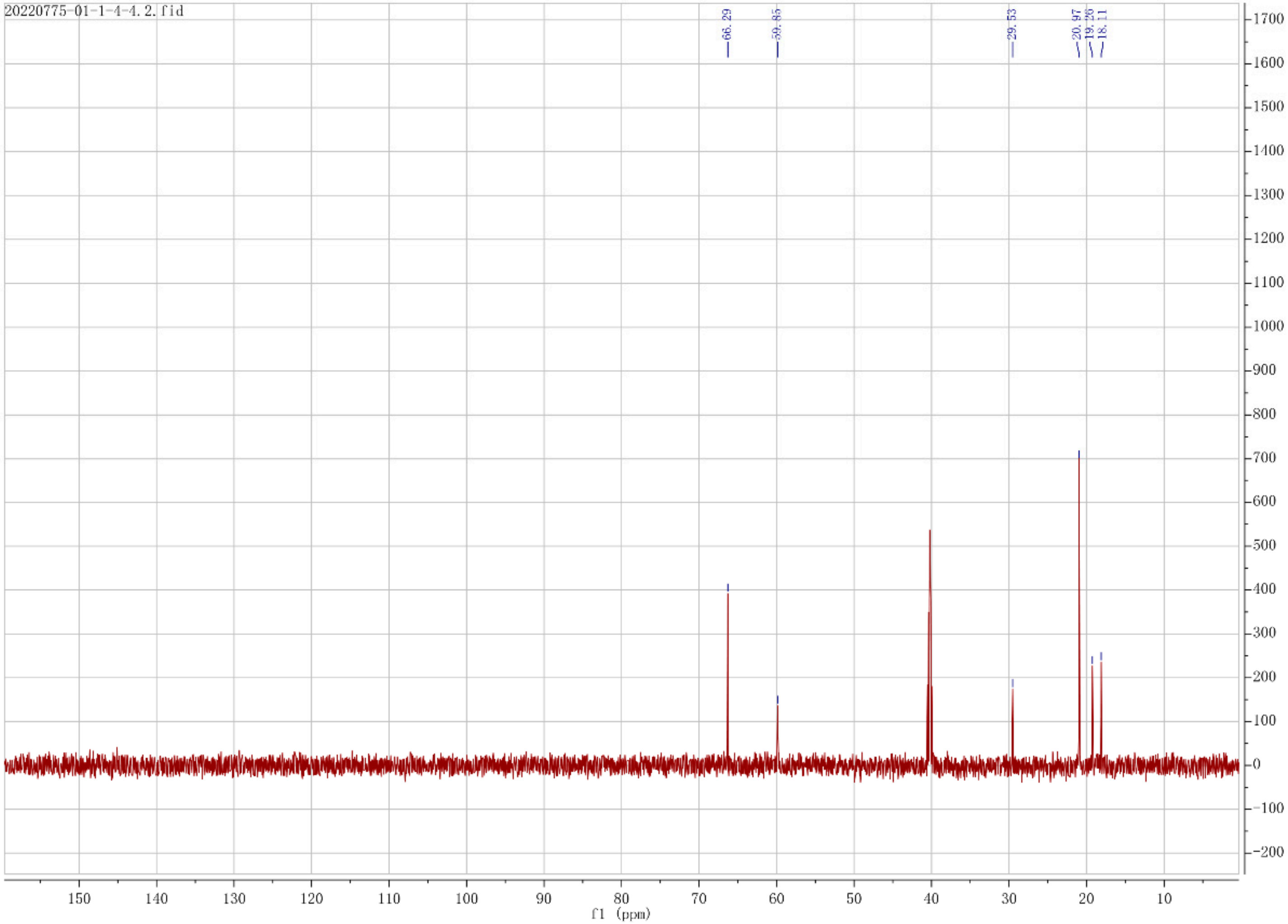

Figure S11. DEPT spectrum of **1-1-4-3** in DMSO-d_6

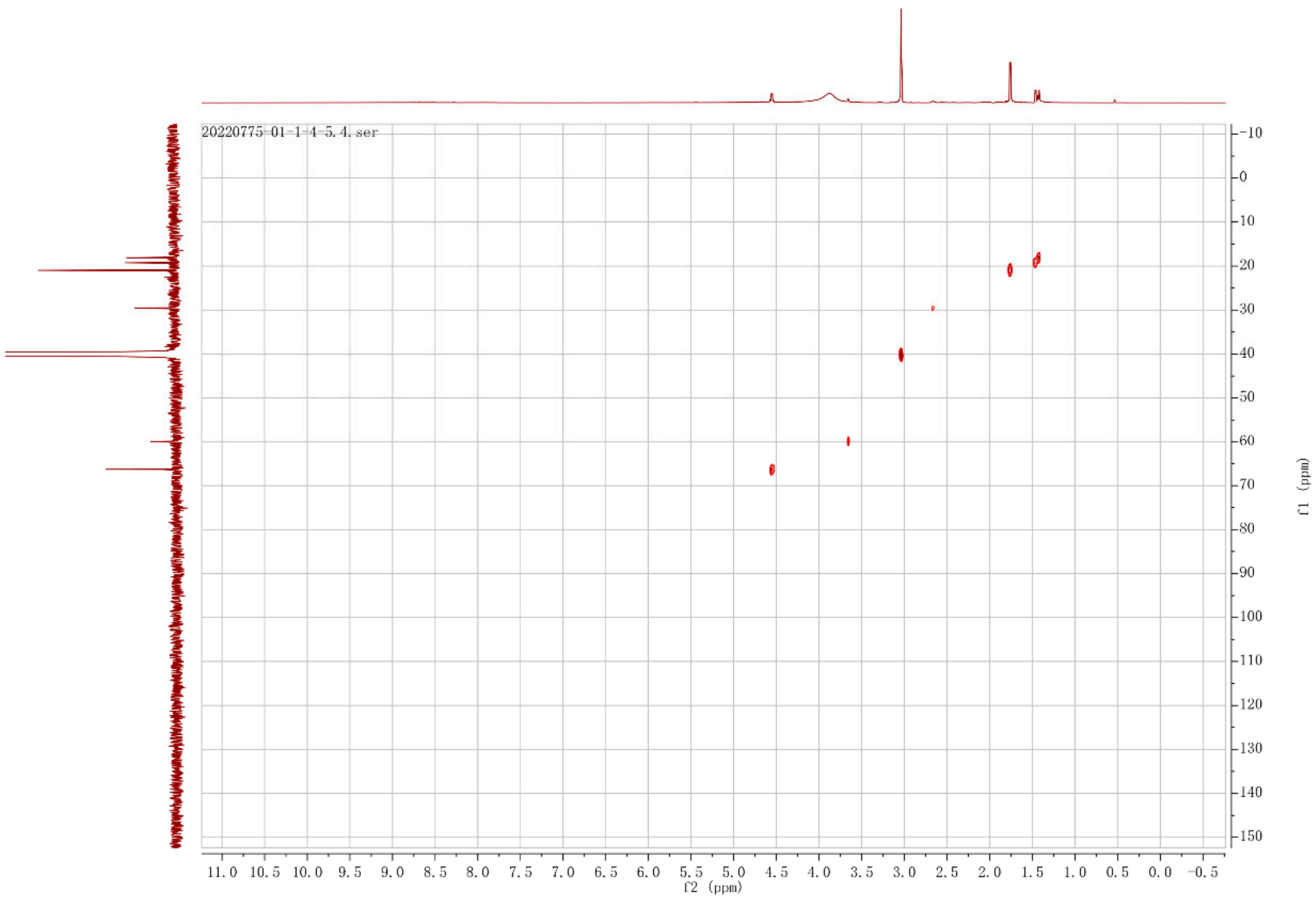
20220775-01-1-4-5.4.ser
f2 (ppm)
f1 (ppm)
11.0 10.5 10.0 9.5 9.0 8.5 8.0 7.5 7.0 6.5 6.0 5.5 5.0 4.5 4.0 3.5 3.0 2.5 2.0 1.5 1.0 0.5 0.0 -0.5
-10 0 10 20 30 40 50 60 70 80 90 100 110 120 130 140 150

Figure S12. HSQC spectrum of **1-1-4-3** in DMSO-d_6

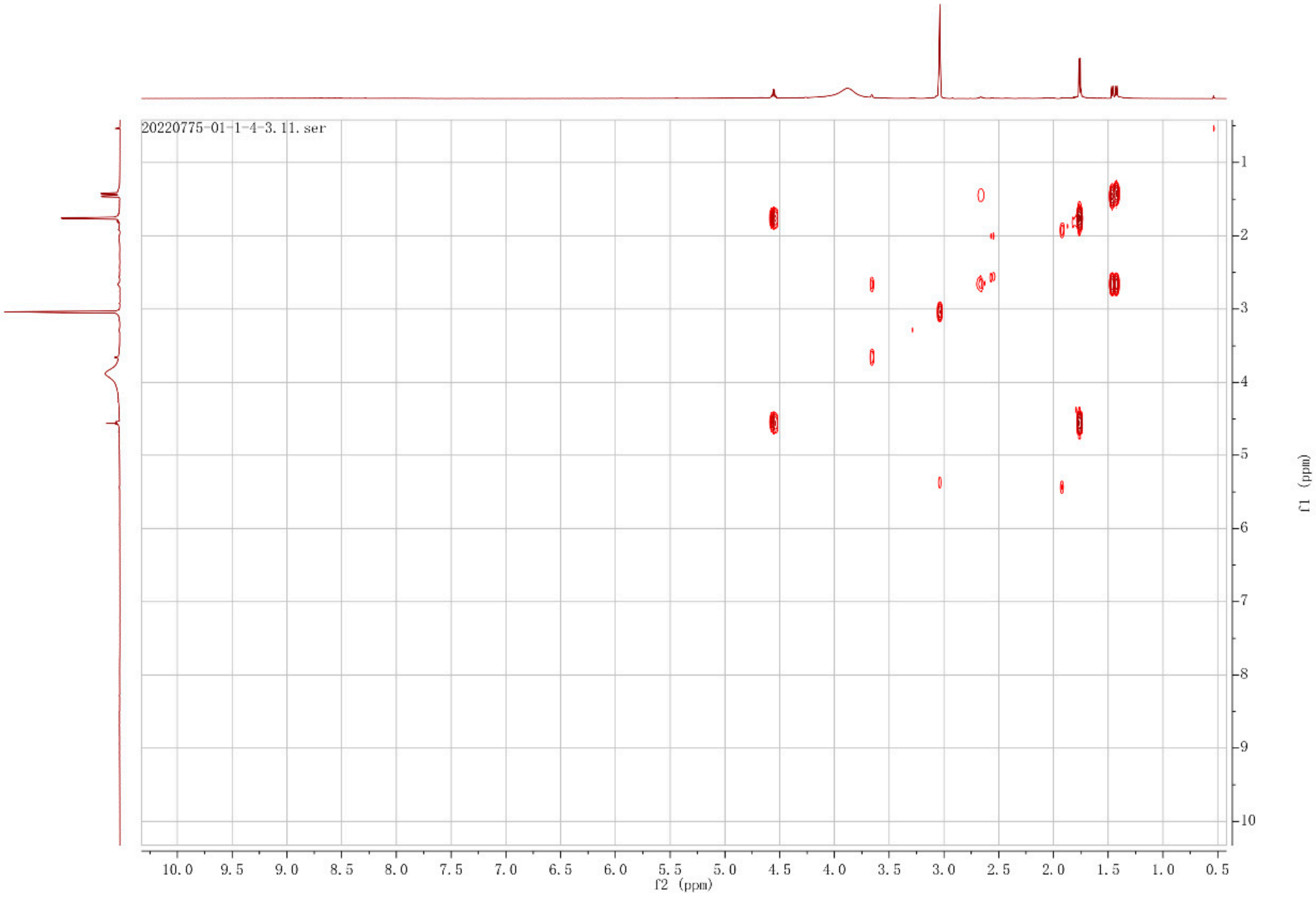

Figure S13. ^{1}H-^{1}H COSY spectrum of **1-1-4-3** in DMSO-d_6

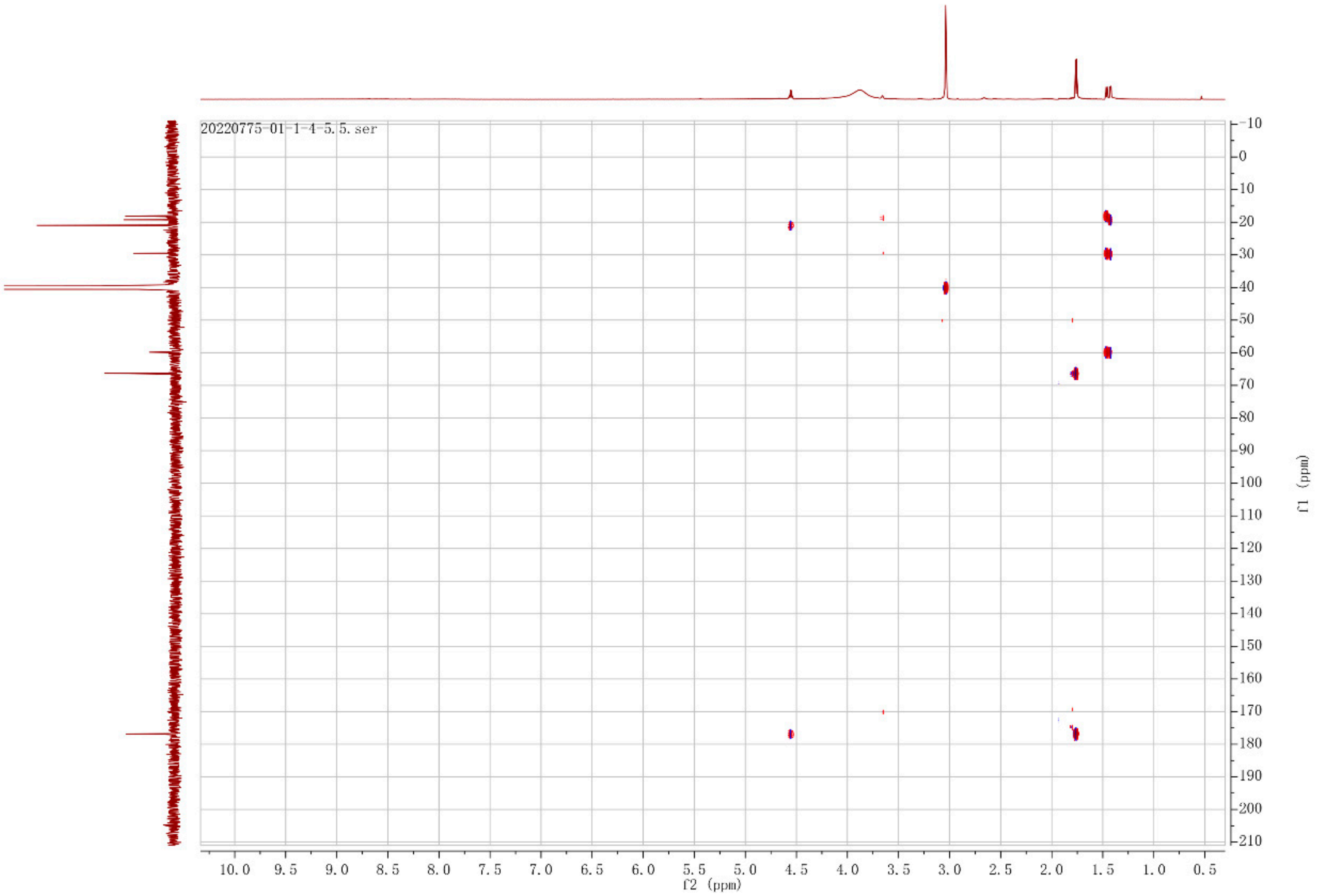

Figure S14. HMBC Spectrum of **1-1-4-3** in DMSO-d_6

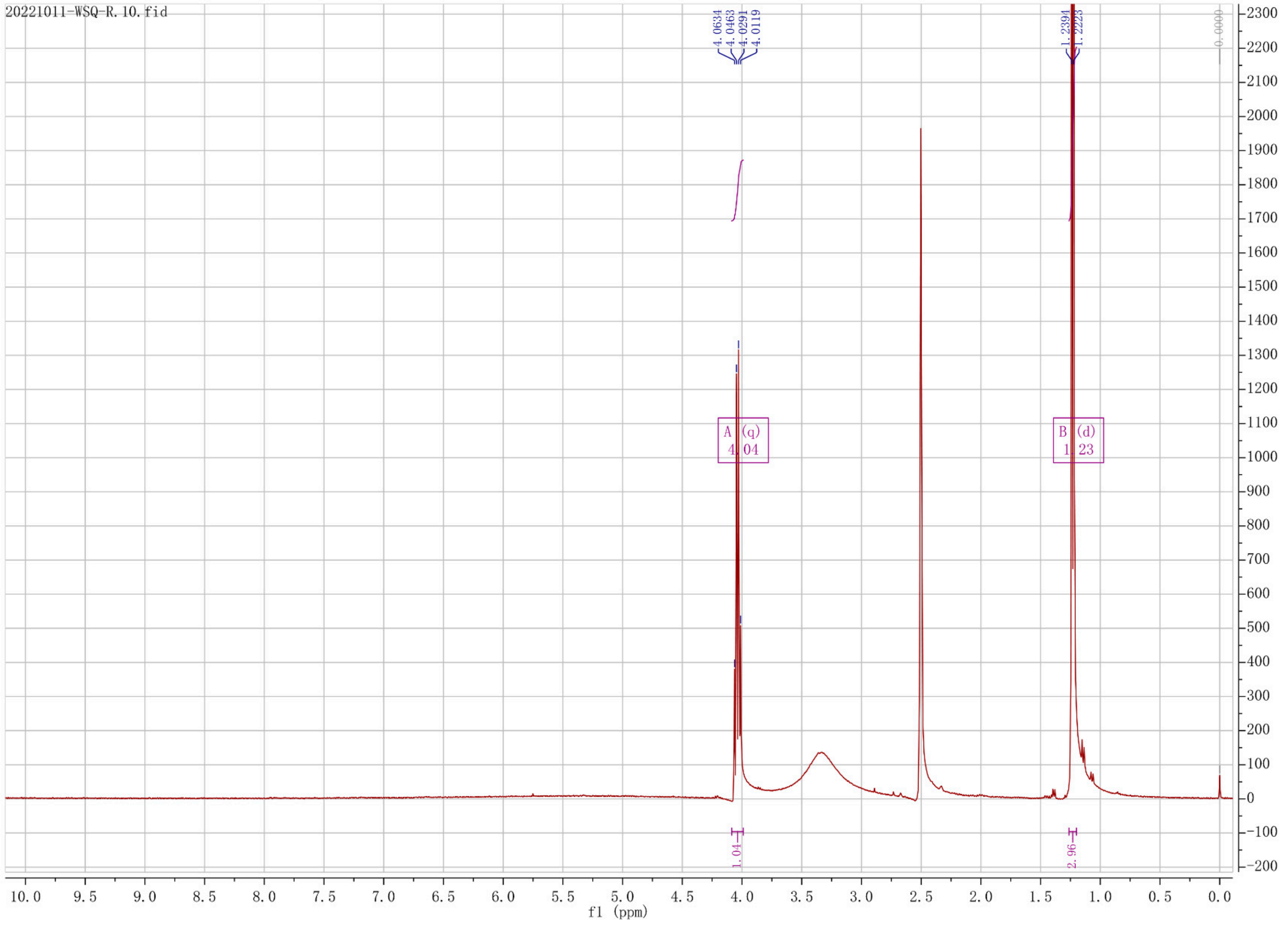
20221011-WSQ-R.10.fid
4.0634
4.0463
4.0291
4.0119
1.2394
1.2223
0.0000
A (q)
4.04
B (d)
1.23
1.04
2.96
f1 (ppm)

Figure S15. ^{1}H NMR spectrum of LA in DMSO-d_6

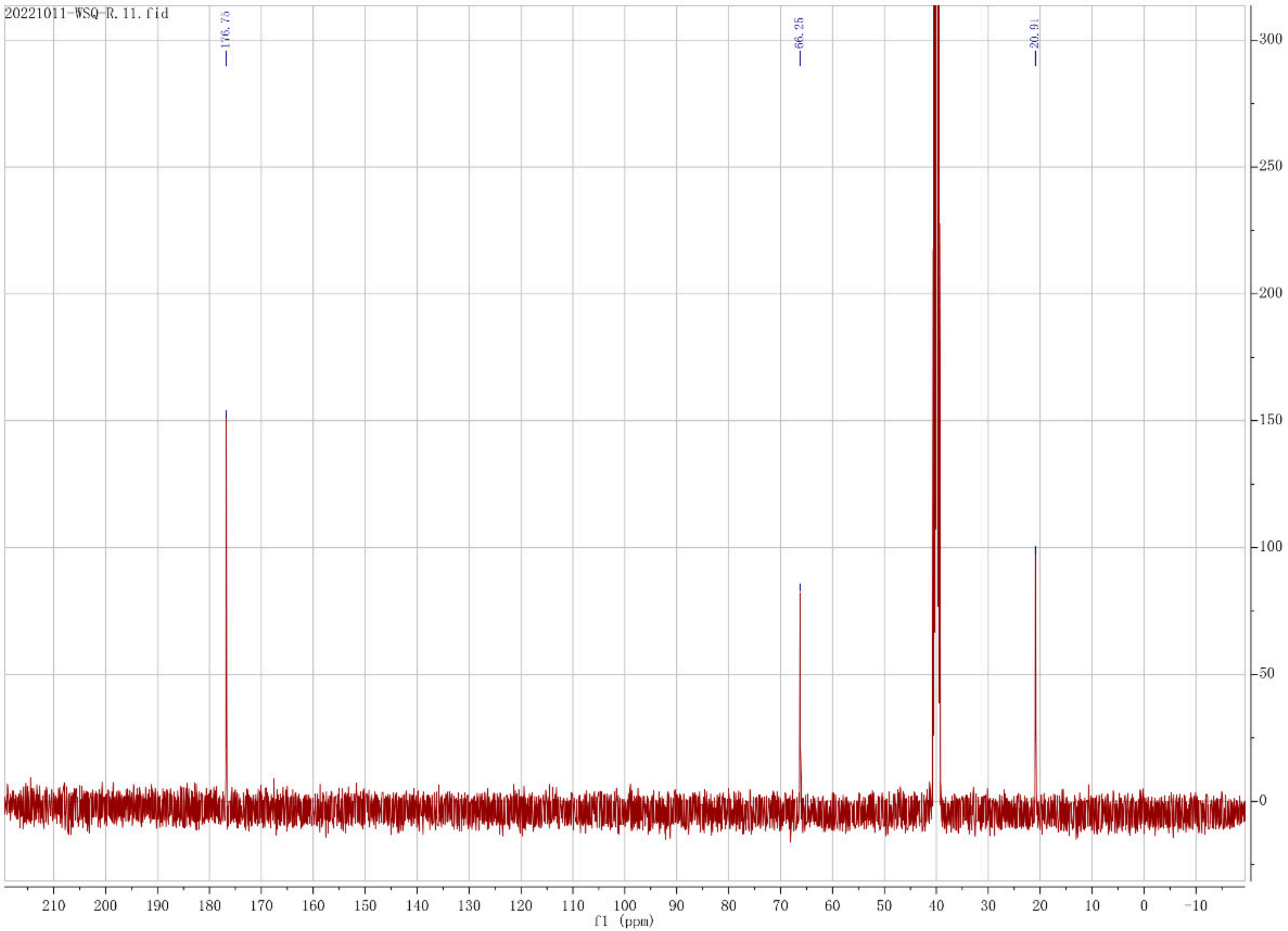

Figure S16. ^{13}C NMR spectrum of LA in DMSO-d_6

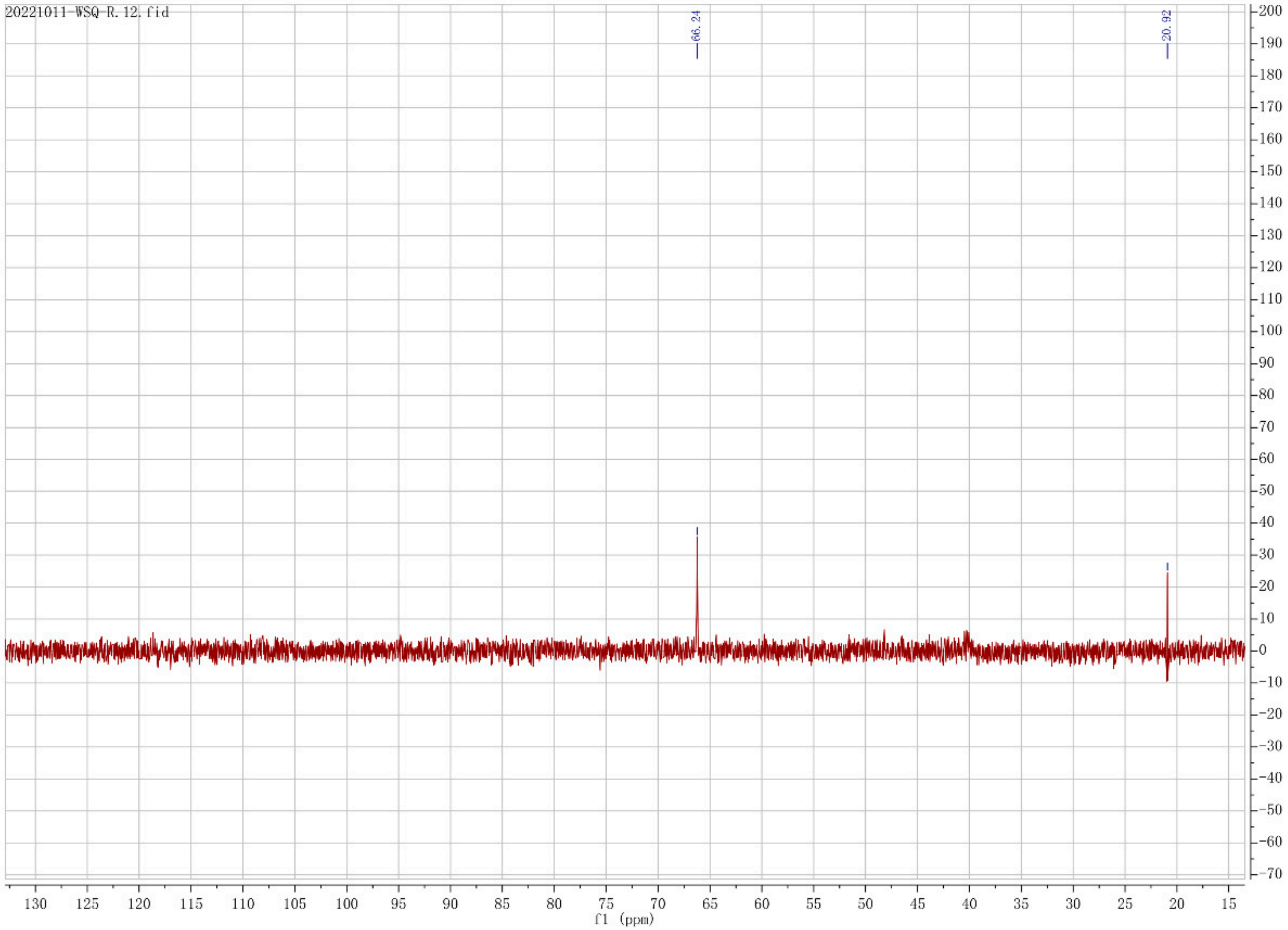

Figure S17. DEPT spectrum of LA in DMSO-d_6

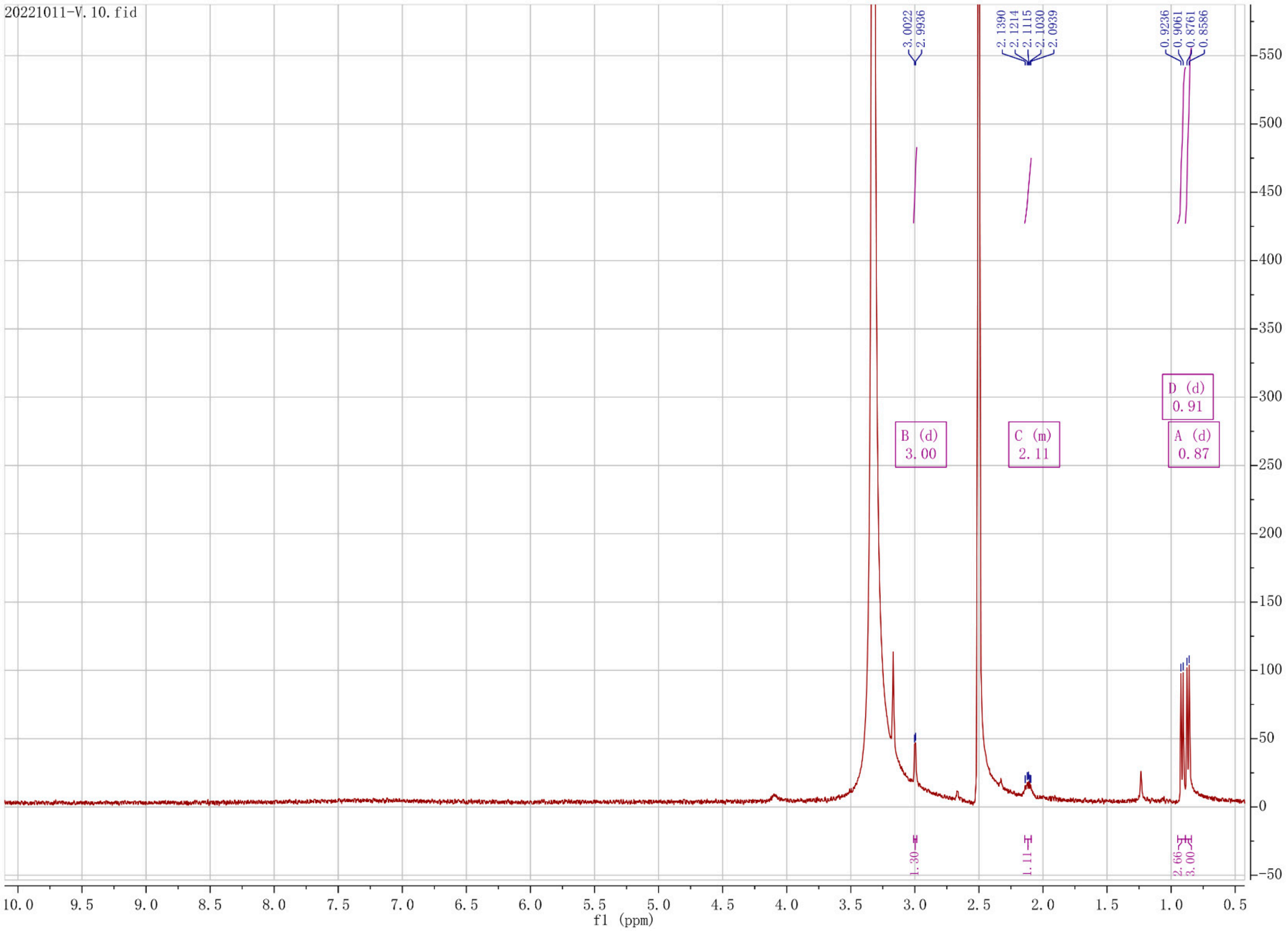

Figure S18. ^{1}H NMR spectrum of valine in DMSO-d_6

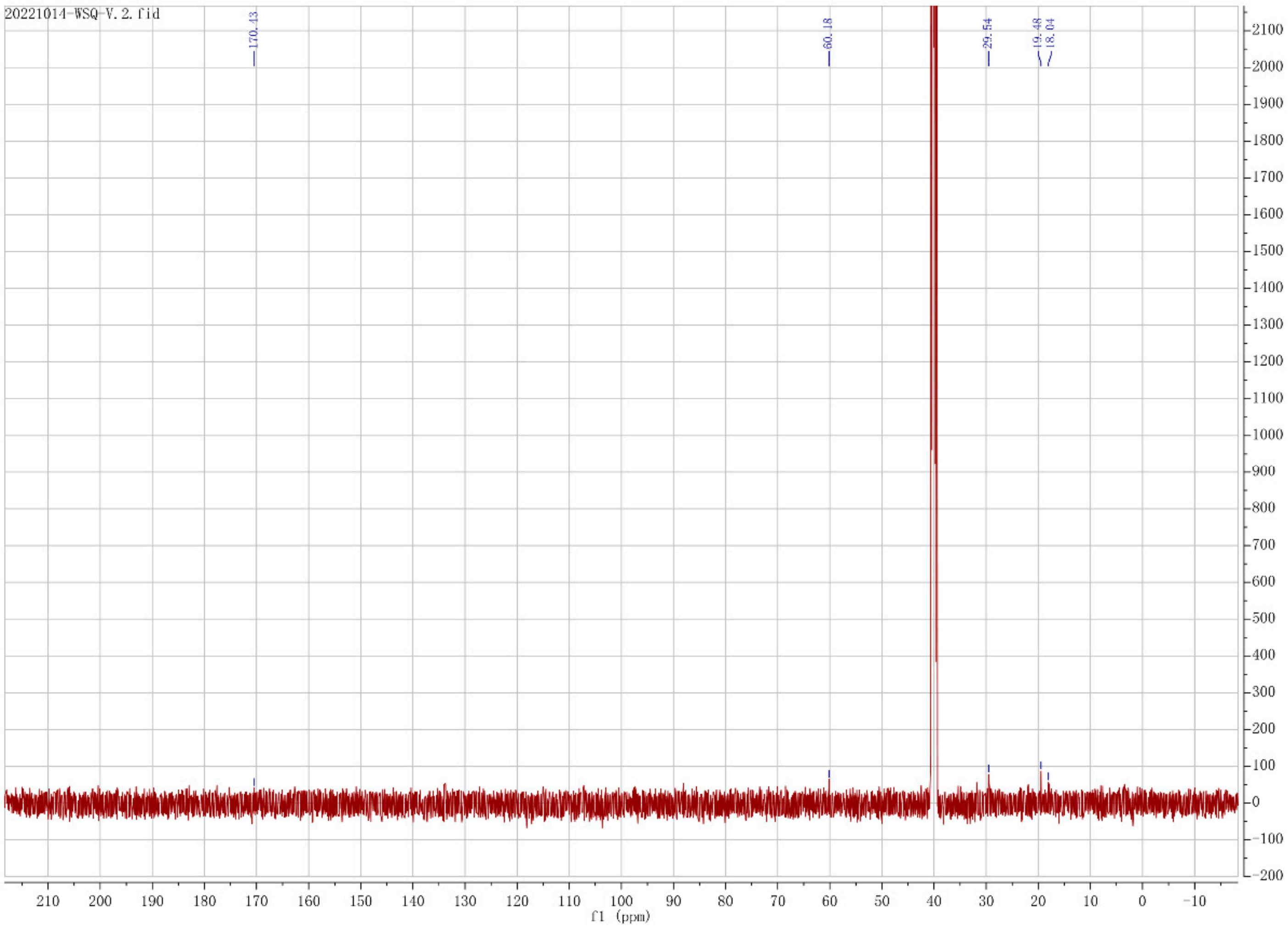

Figure S19. ^{13}C NMR spectrum of valine in DMSO-d_6

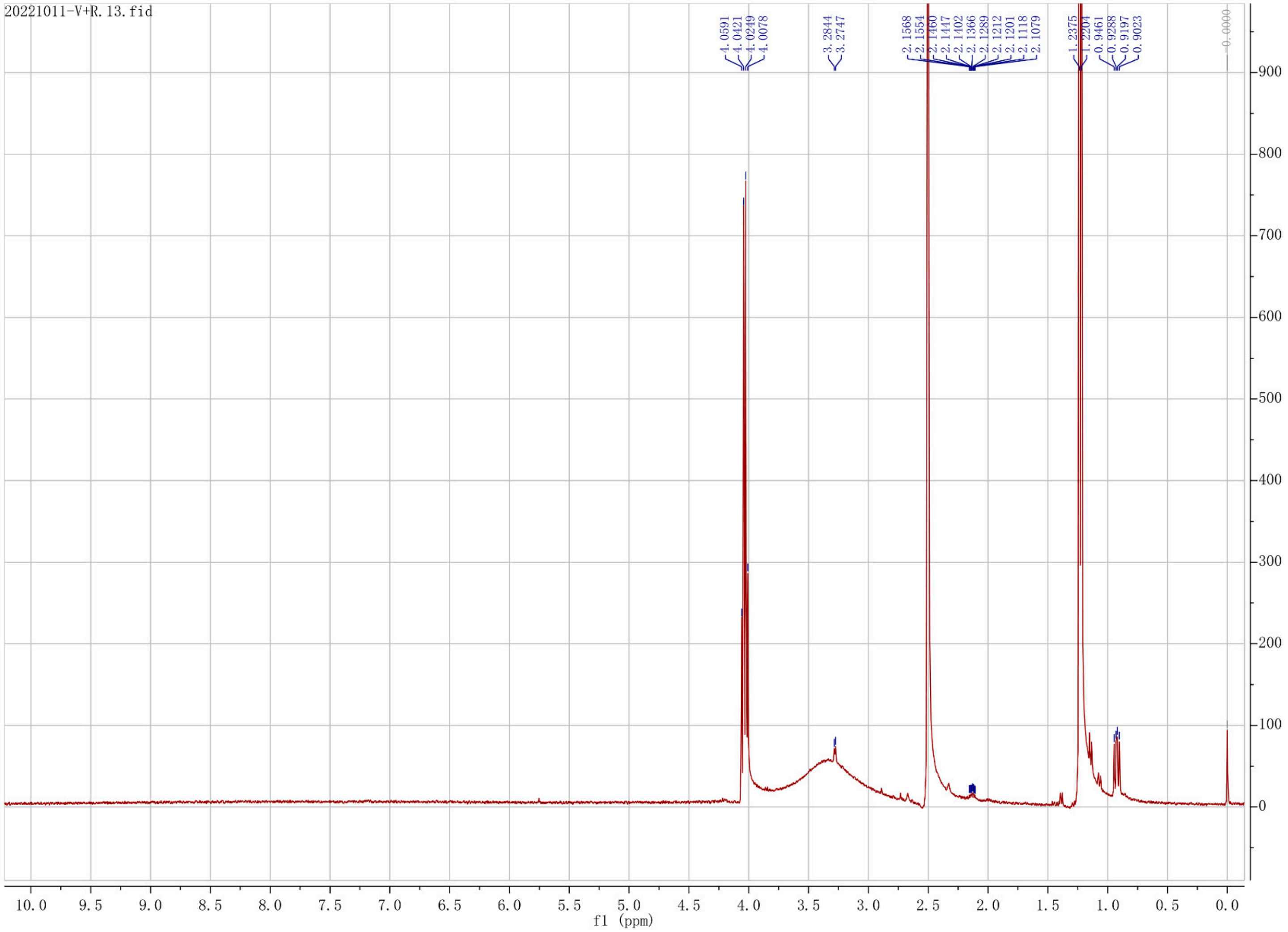

Figure S20. ^{1}H NMR spectrum of mixture of valine and LA in DMSO-d_6

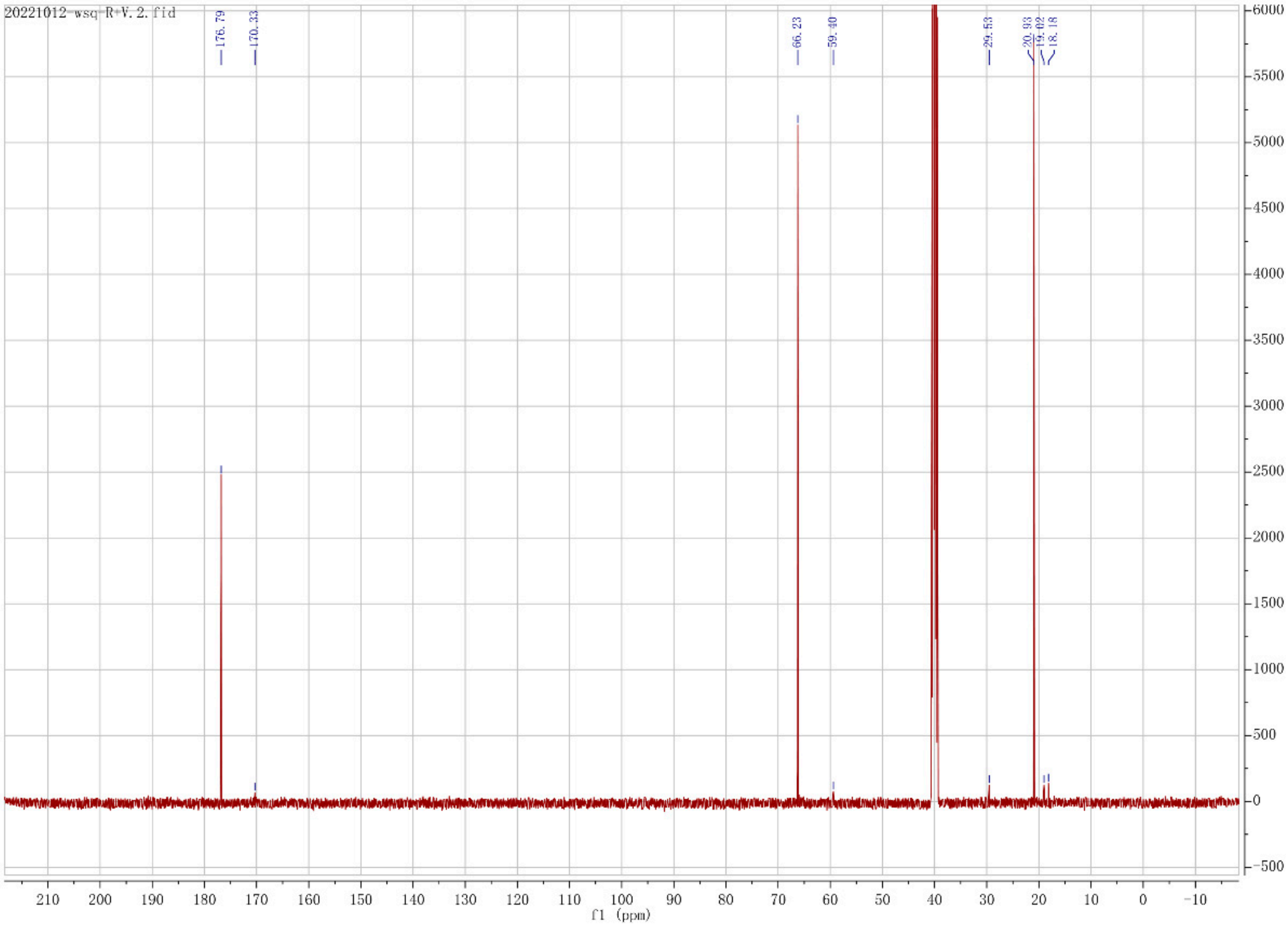

Figure S21. ^{13}C NMR spectrum of mixture of valine and LA in DMSO-d_6

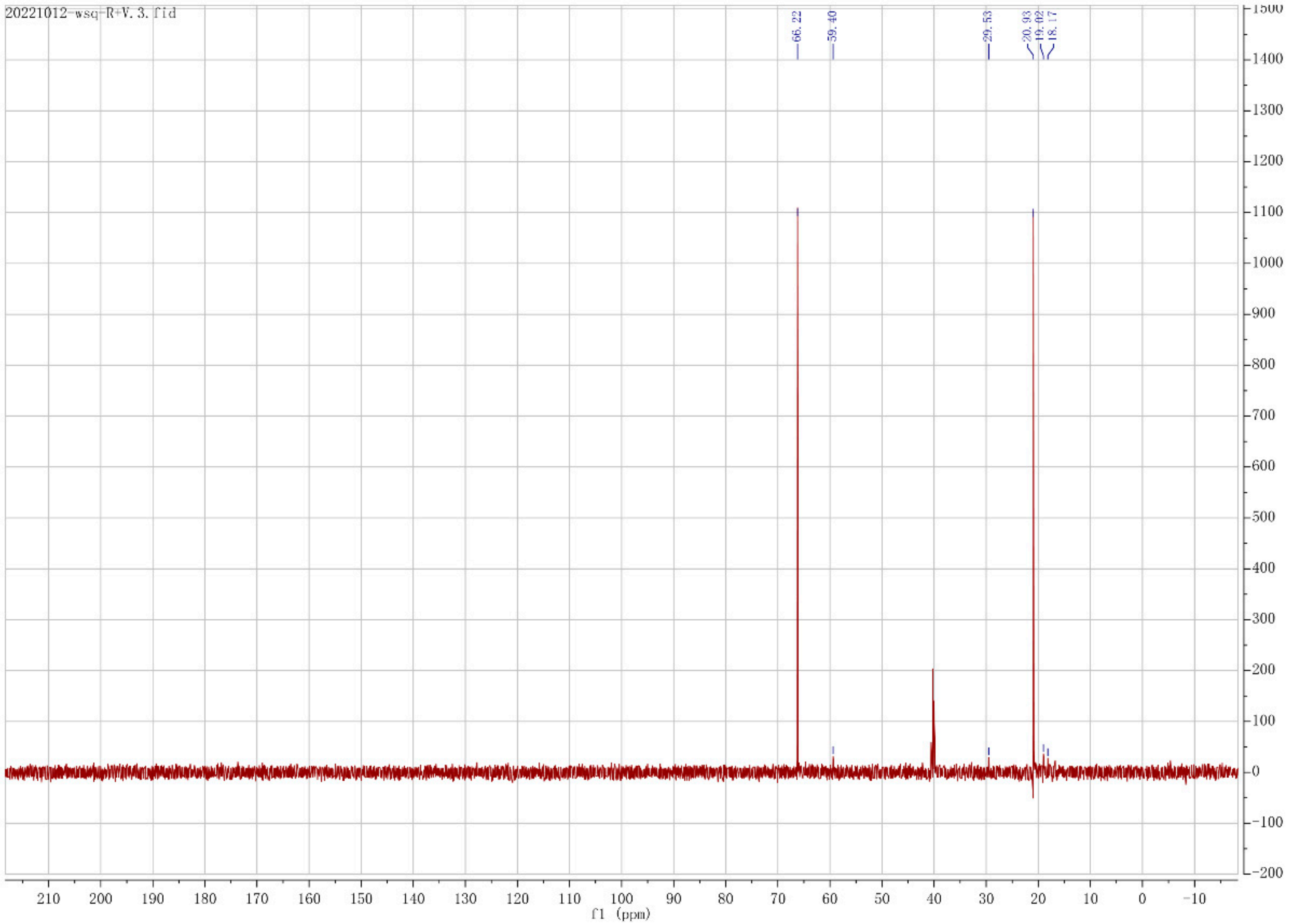

Figure S22. DEPT spectrum of mixture of valine and LA in DMSO-d_6
