# Supplementary figures and images for: Exploration of the Main Antibiofilm Substance of Lactobacillus plantarum ATCC 14917 and Its Effect against Streptococcus mutans
Source: Int J Mol Sci. 2023 Jan 19;24(3):1986. doi: 10.3390/ijms24031986 (PMC9916977; doi:10.3390/ijms24031986)

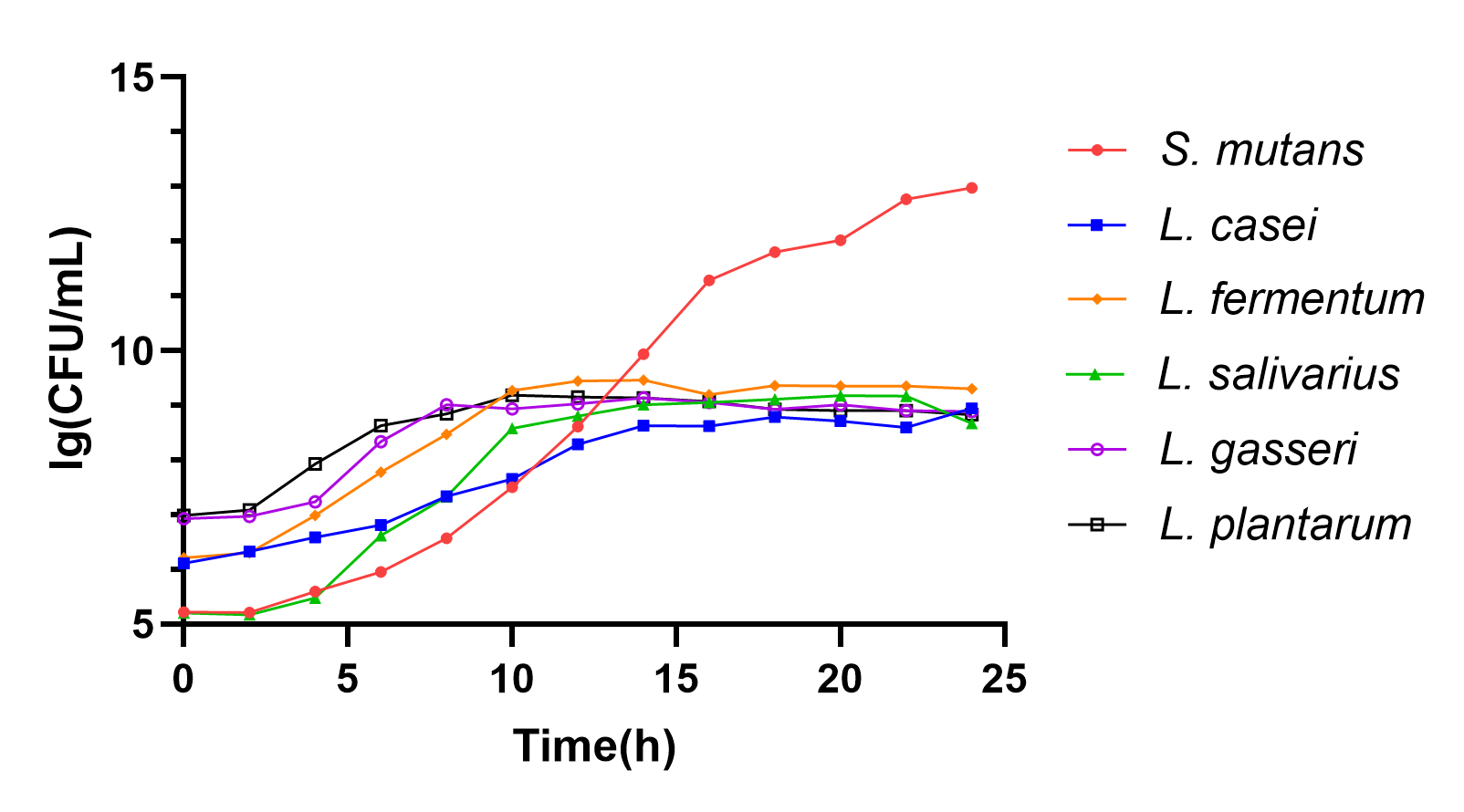

Supplement: Supplementary file 1 [file ijms-24-01986-s001.zip › Figure S1. Growth curves of S. mutans and five selected Lactobacilli.tif]

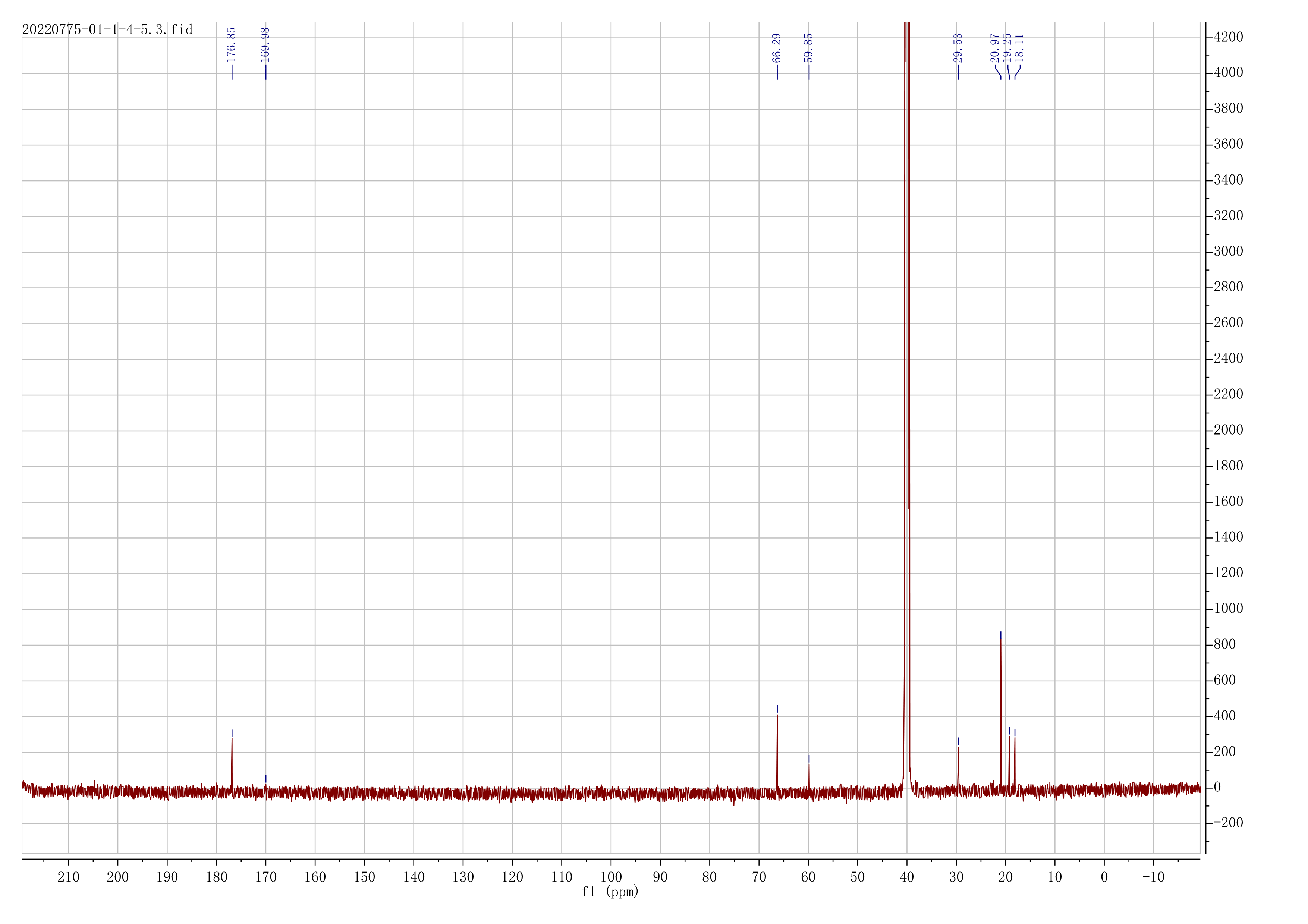

Supplement: Supplementary file 1 [file ijms-24-01986-s001.zip › Figure S10. 13C NMR spectrum of 1-1-4-3 in DMSO-d6.tif]

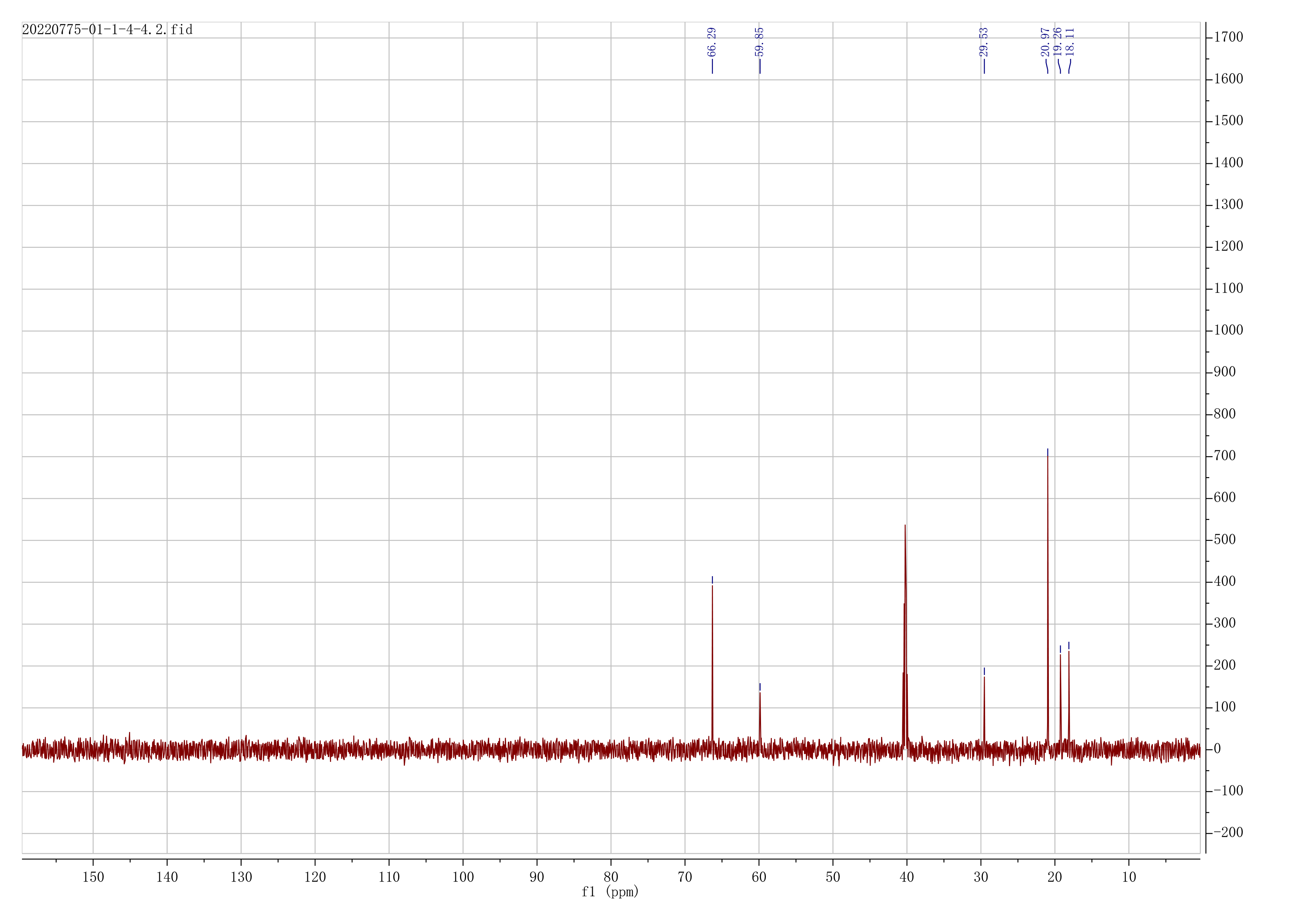

Supplement: Supplementary file 1 [file ijms-24-01986-s001.zip › Figure S11. DEPT spectrum of 1-1-4-3 in DMSO-d6.tif]

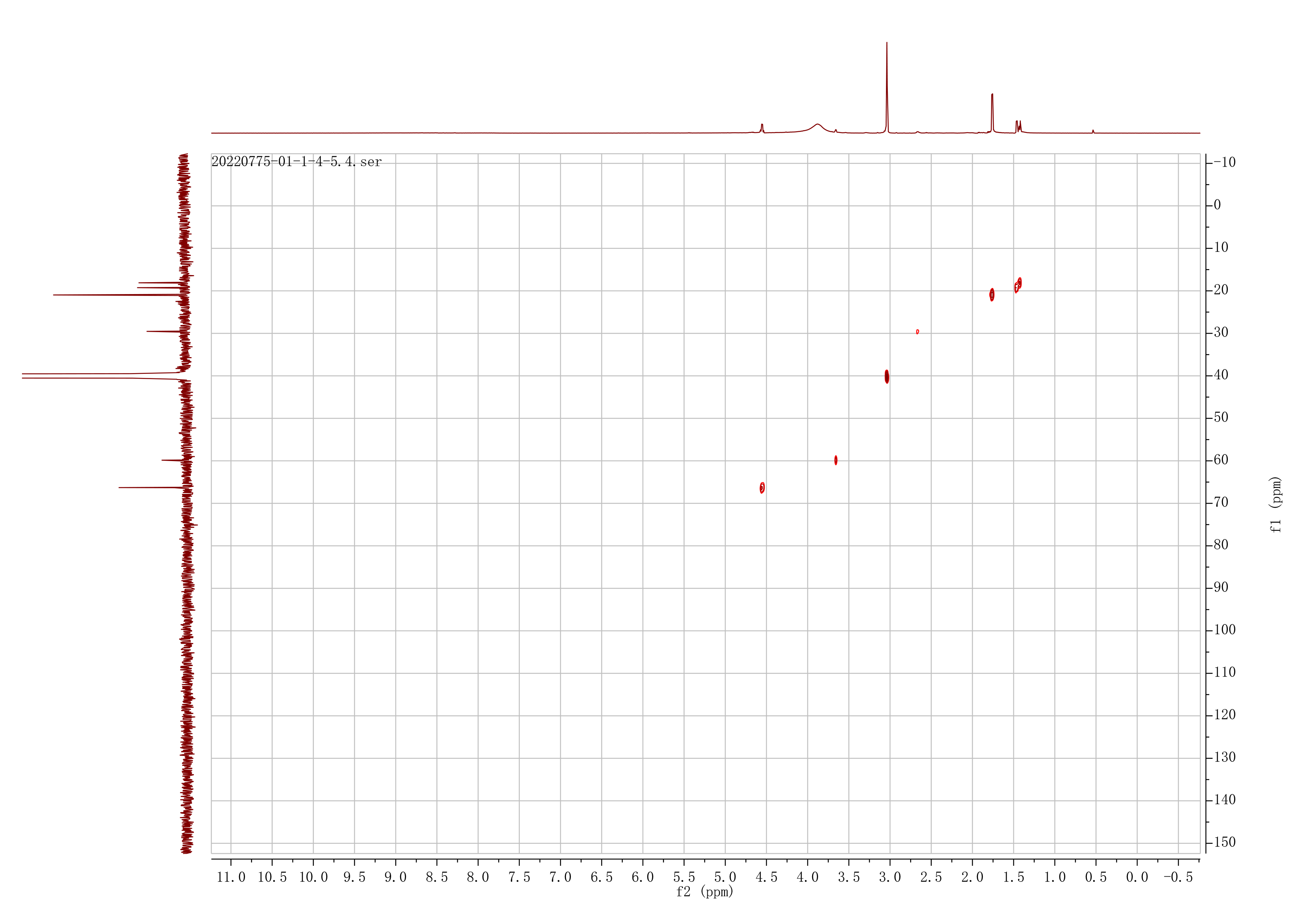

Supplement: Supplementary file 1 [file ijms-24-01986-s001.zip › Figure S12. HSQC spectrum of 1-1-4-3 in DMSO-d6.tif]

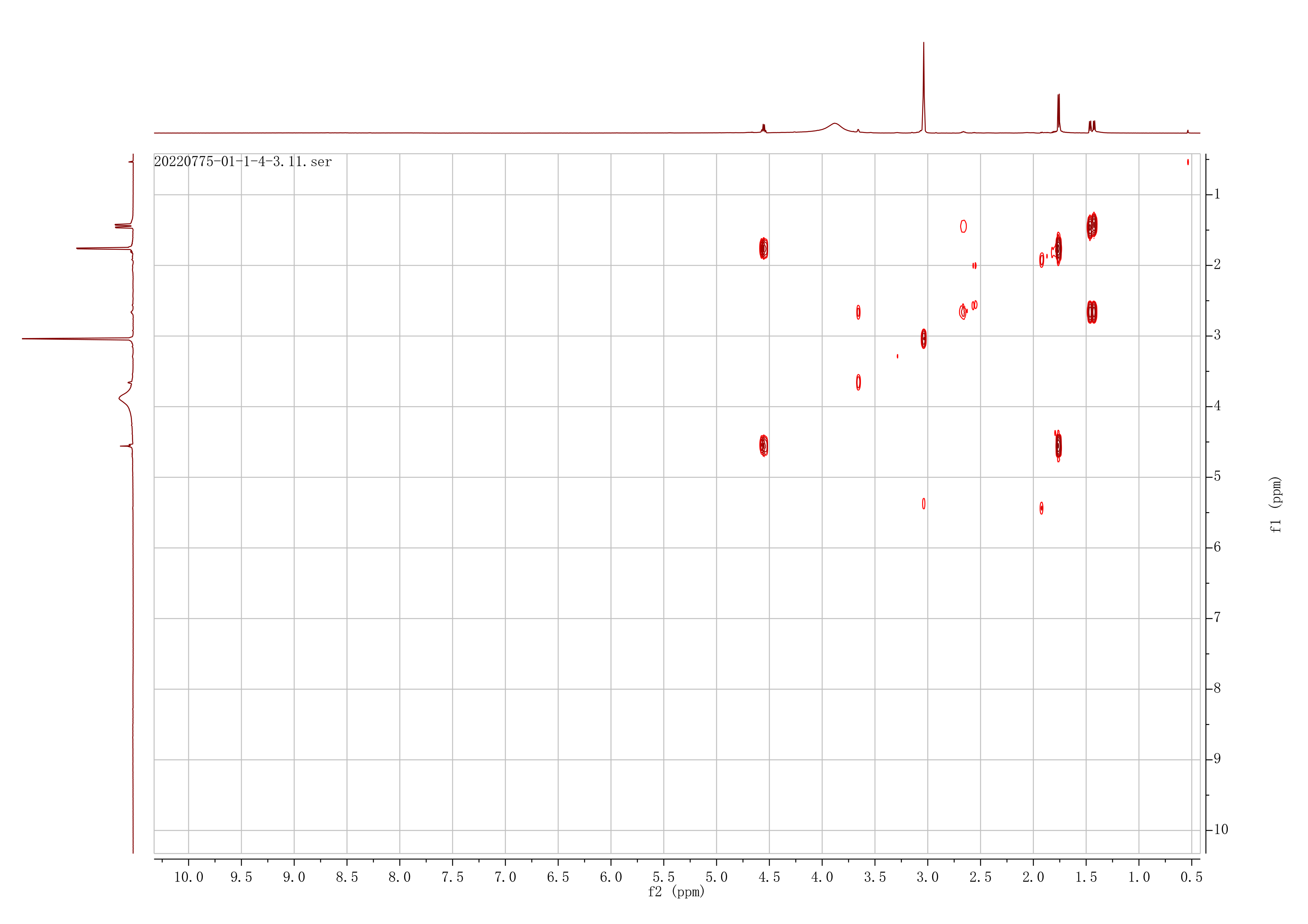

Supplement: Supplementary file 1 [file ijms-24-01986-s001.zip › Figure S13. 1H-1H COSY spectrum of 1-1-4-3 in DMSO-d6.tif]

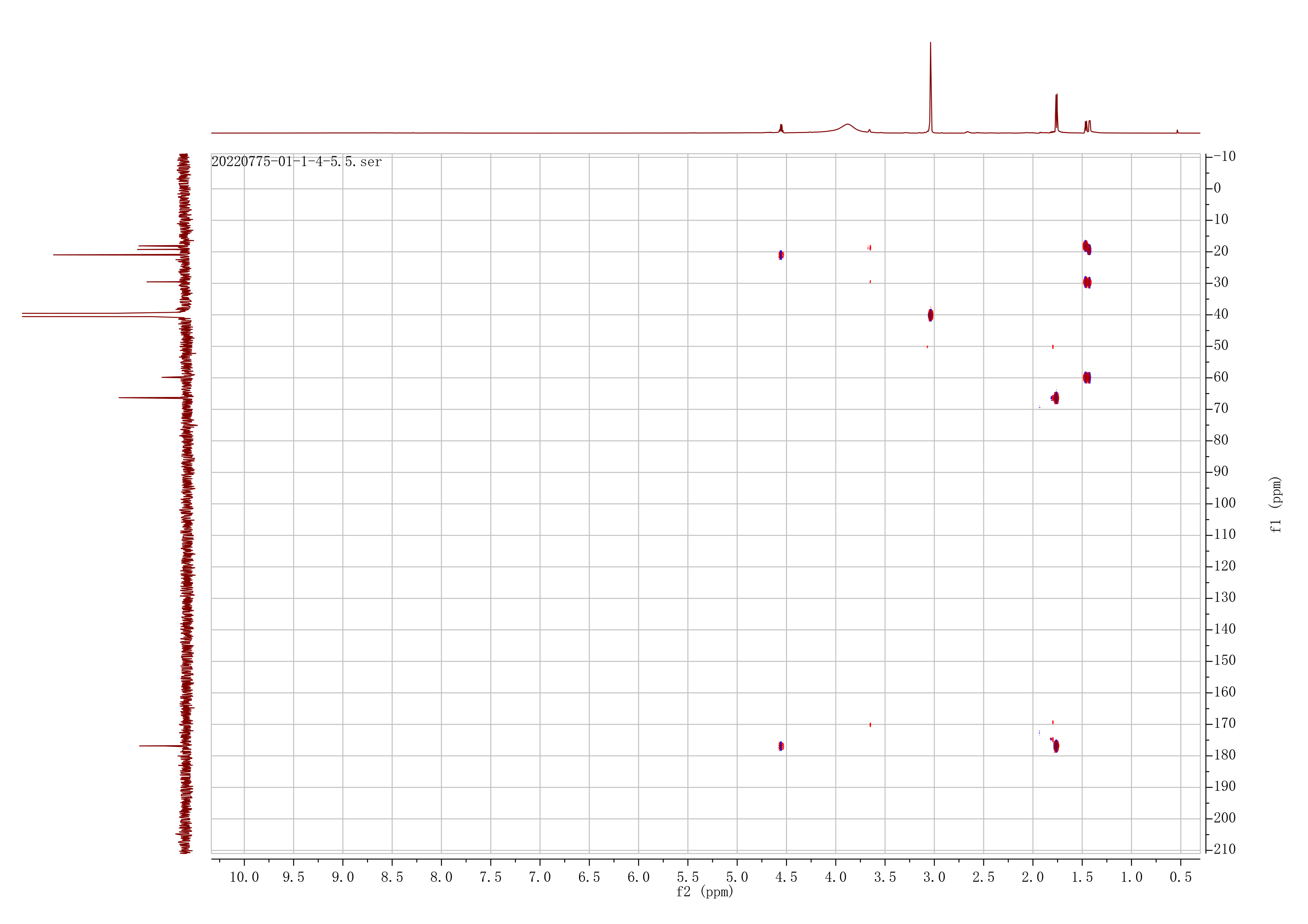

Supplement: Supplementary file 1 [file ijms-24-01986-s001.zip › Figure S14. HMBC Spectrum of 1-1-4-3 in DMSO-d6.tif]

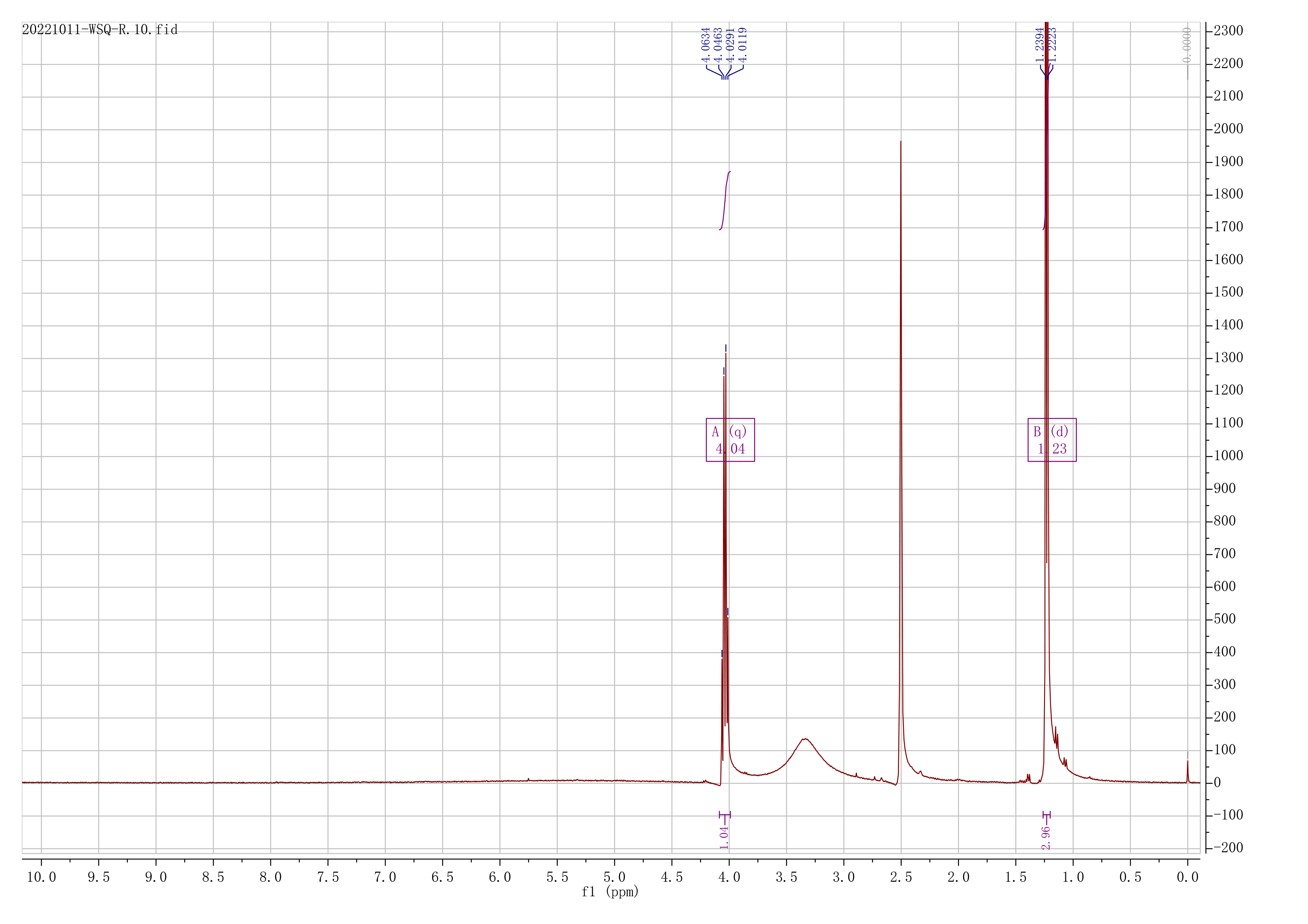

Supplement: Supplementary file 1 [file ijms-24-01986-s001.zip › Figure S15. 1H NMR spectrum of LA in DMSO-d6.tif]

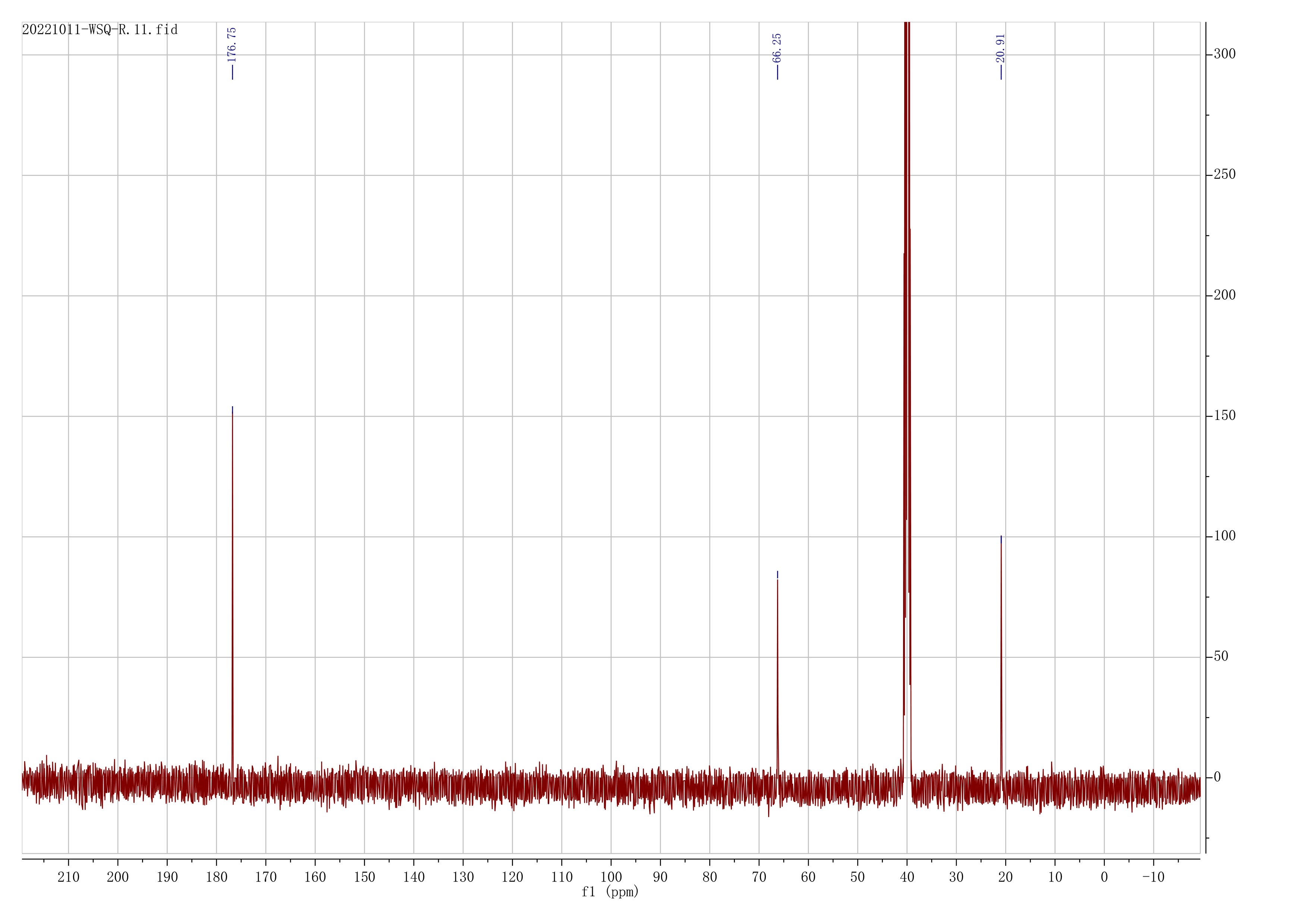

Supplement: Supplementary file 1 [file ijms-24-01986-s001.zip › Figure S16. 13C NMR spectrum of LA in DMSO-d6.tif]

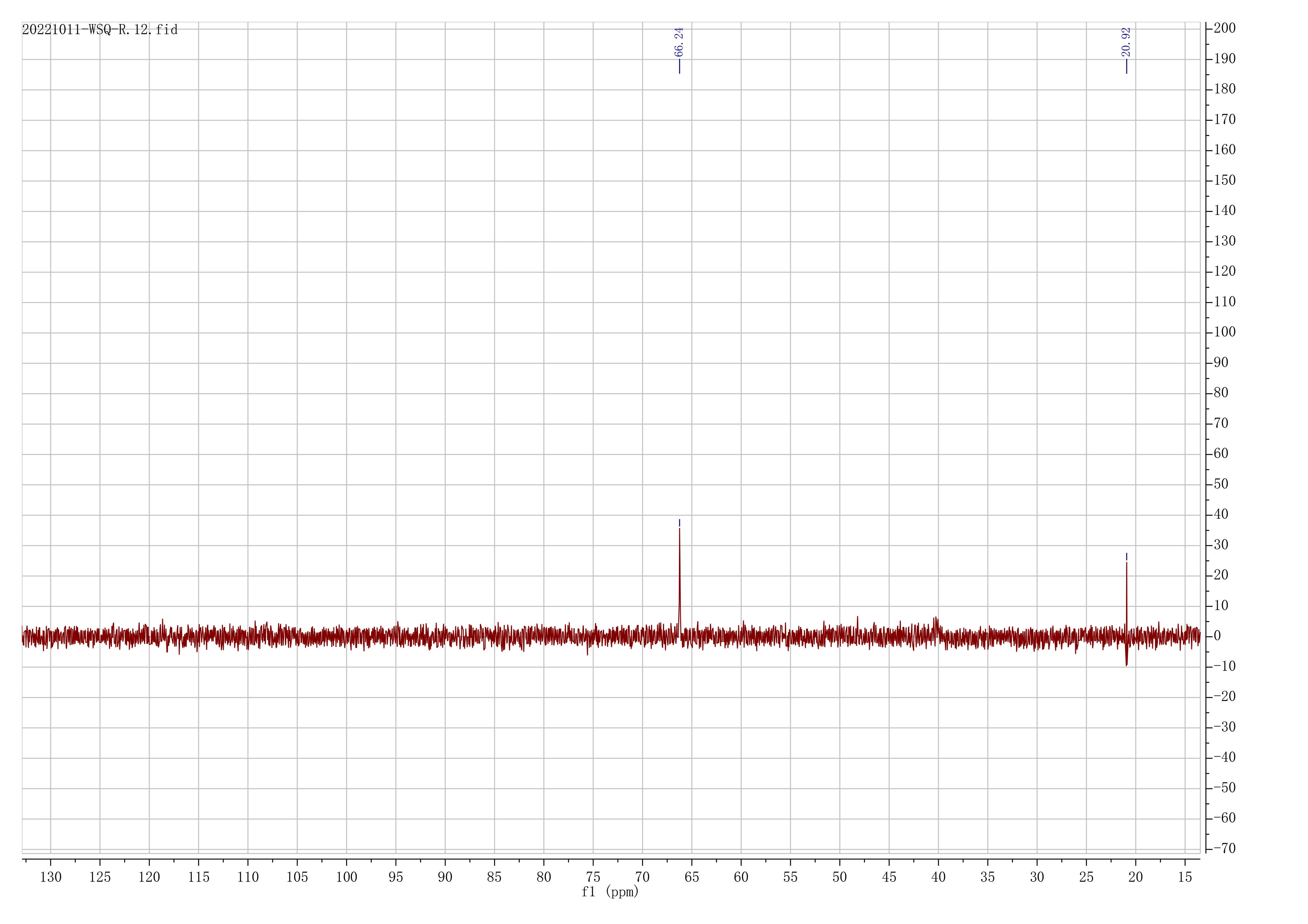

Supplement: Supplementary file 1 [file ijms-24-01986-s001.zip › Figure S17. DEPT spectrum of LA in DMSO-d6.tif]

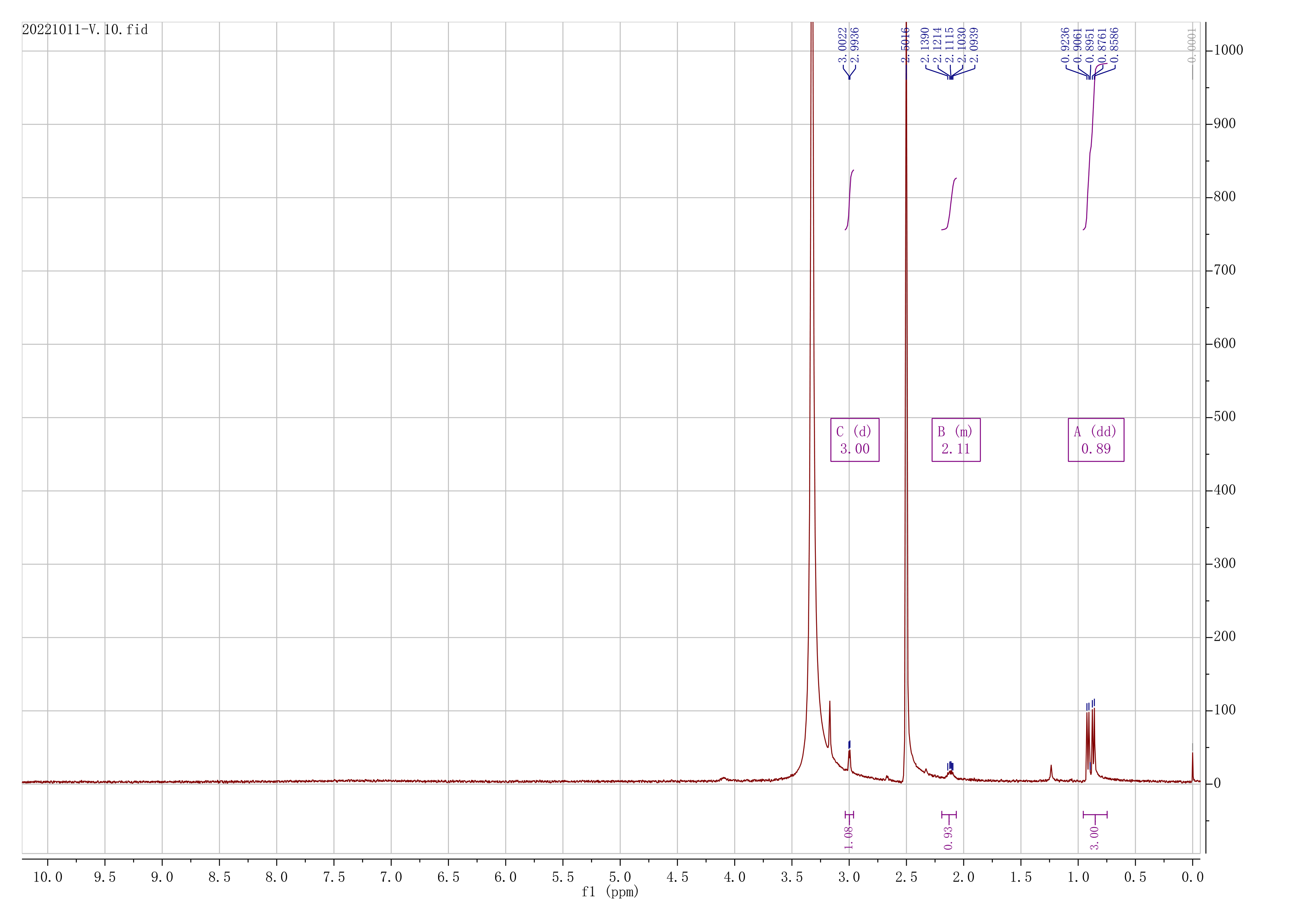

Supplement: Supplementary file 1 [file ijms-24-01986-s001.zip › Figure S18. 1H NMR spectrum of valine in DMSO-d6.tif]

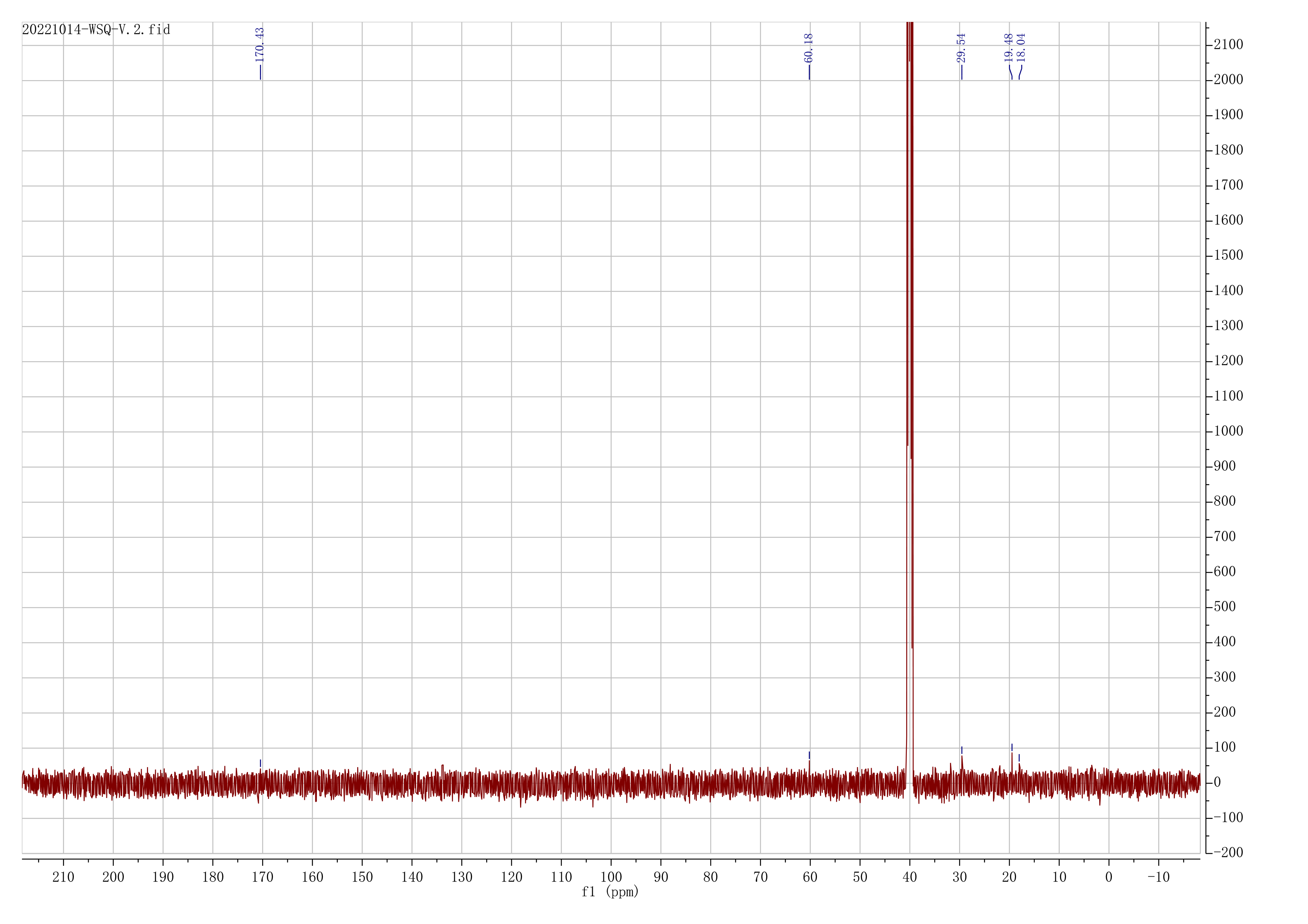

Supplement: Supplementary file 1 [file ijms-24-01986-s001.zip › Figure S19. 13C NMR spectrum of valine in DMSO-d6.tif]

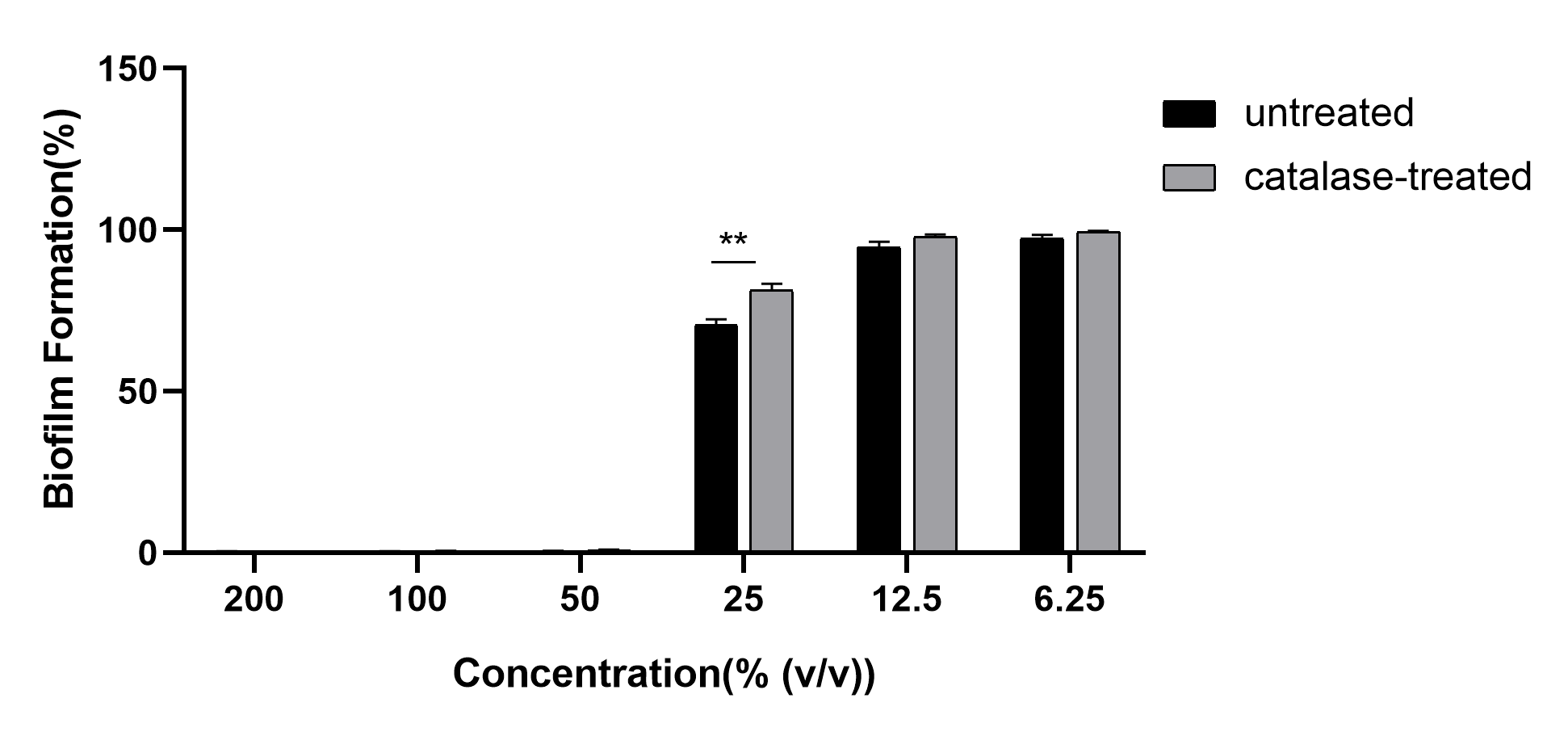

Supplement: Supplementary file 1 [file ijms-24-01986-s001.zip › Figure S2. Biofilm formation of S. mutans in the presence of untreated and catalase-treated CFS of L. salivarius.tif]

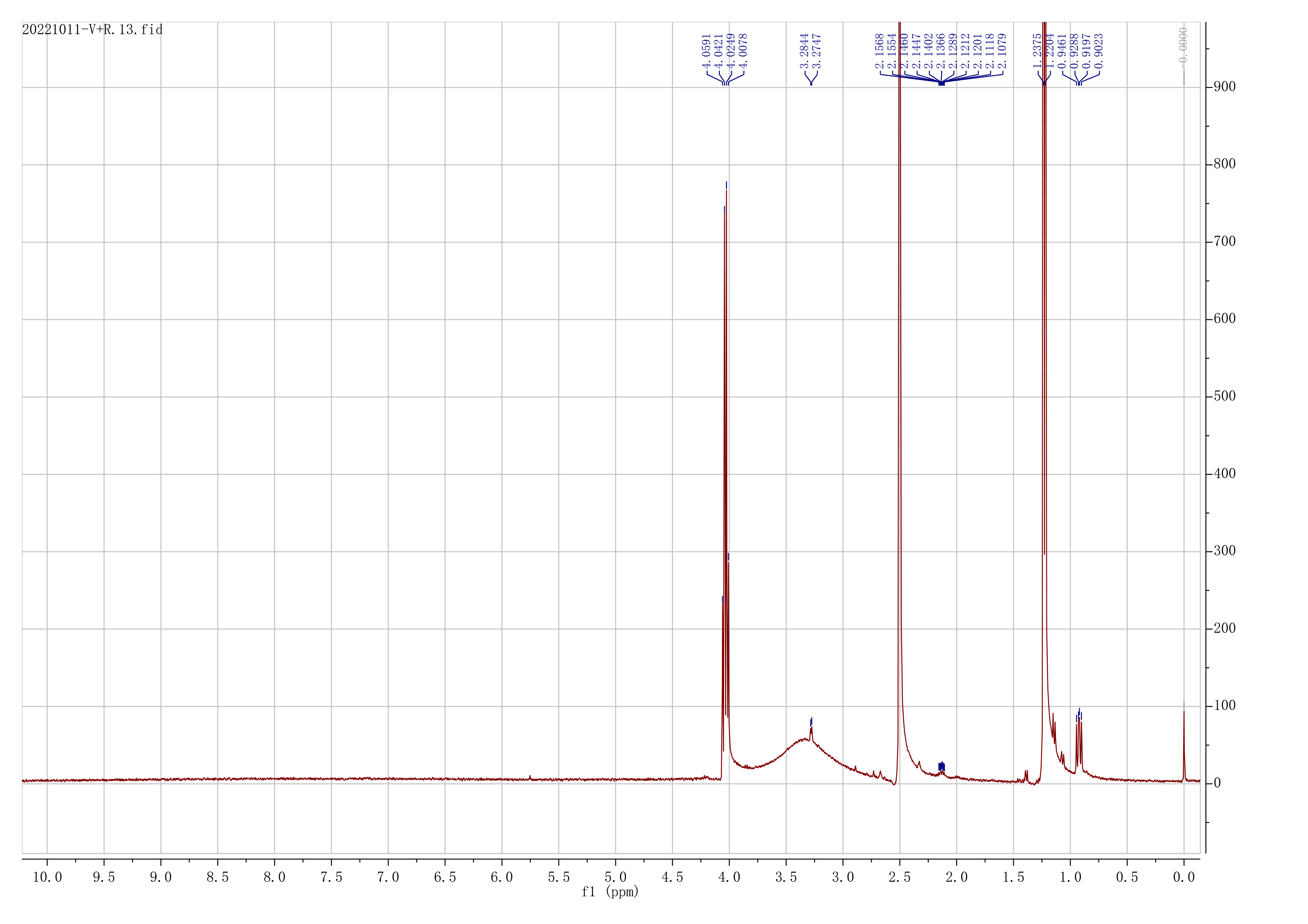

Supplement: Supplementary file 1 [file ijms-24-01986-s001.zip › Figure S20. 1H NMR spectrum of mixture of valine and LA in DMSO-d6.tif]

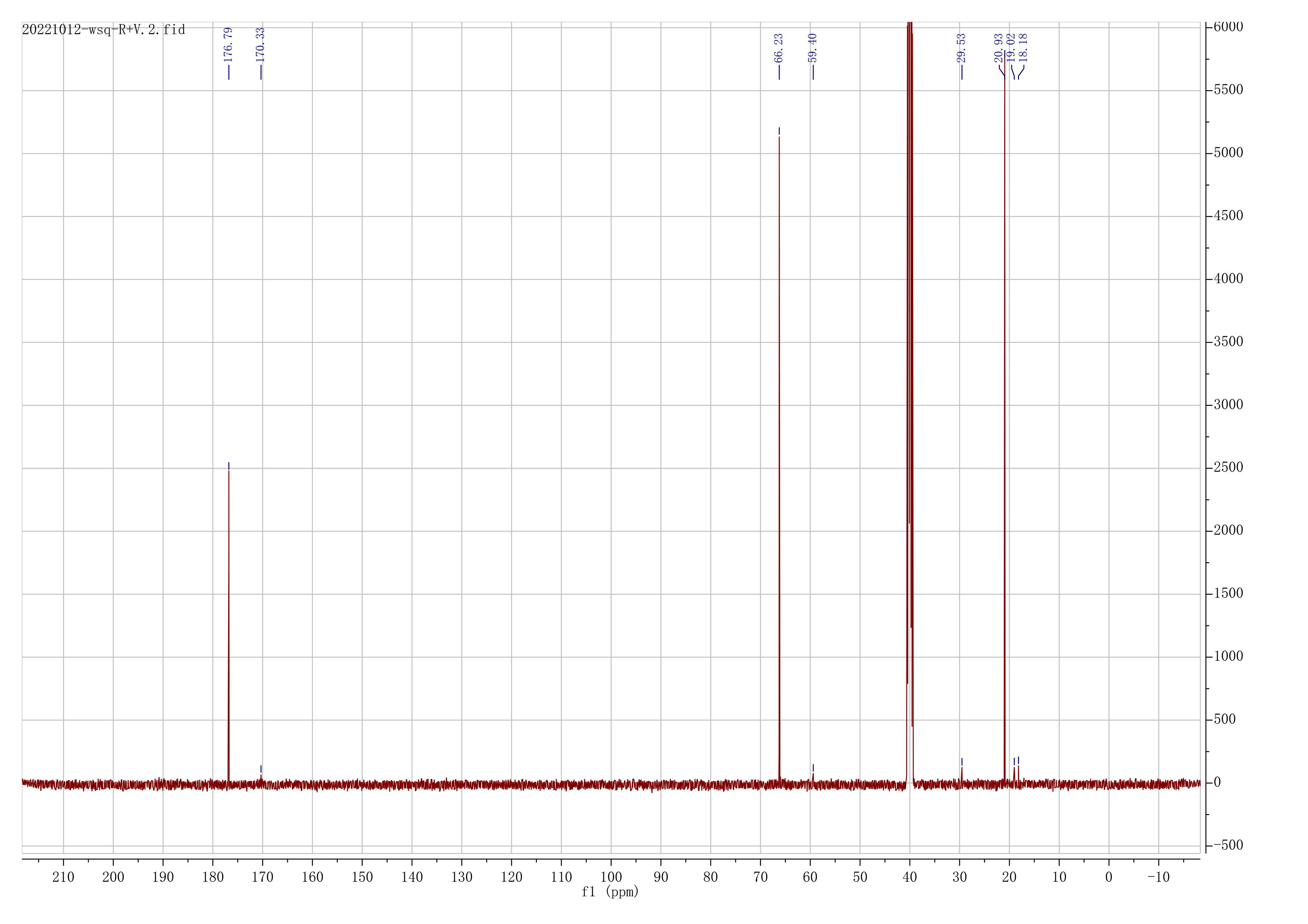

Supplement: Supplementary file 1 [file ijms-24-01986-s001.zip › Figure S21. 13C NMR spectrum of mixture of valine and LA in DMSO-d6.tif]

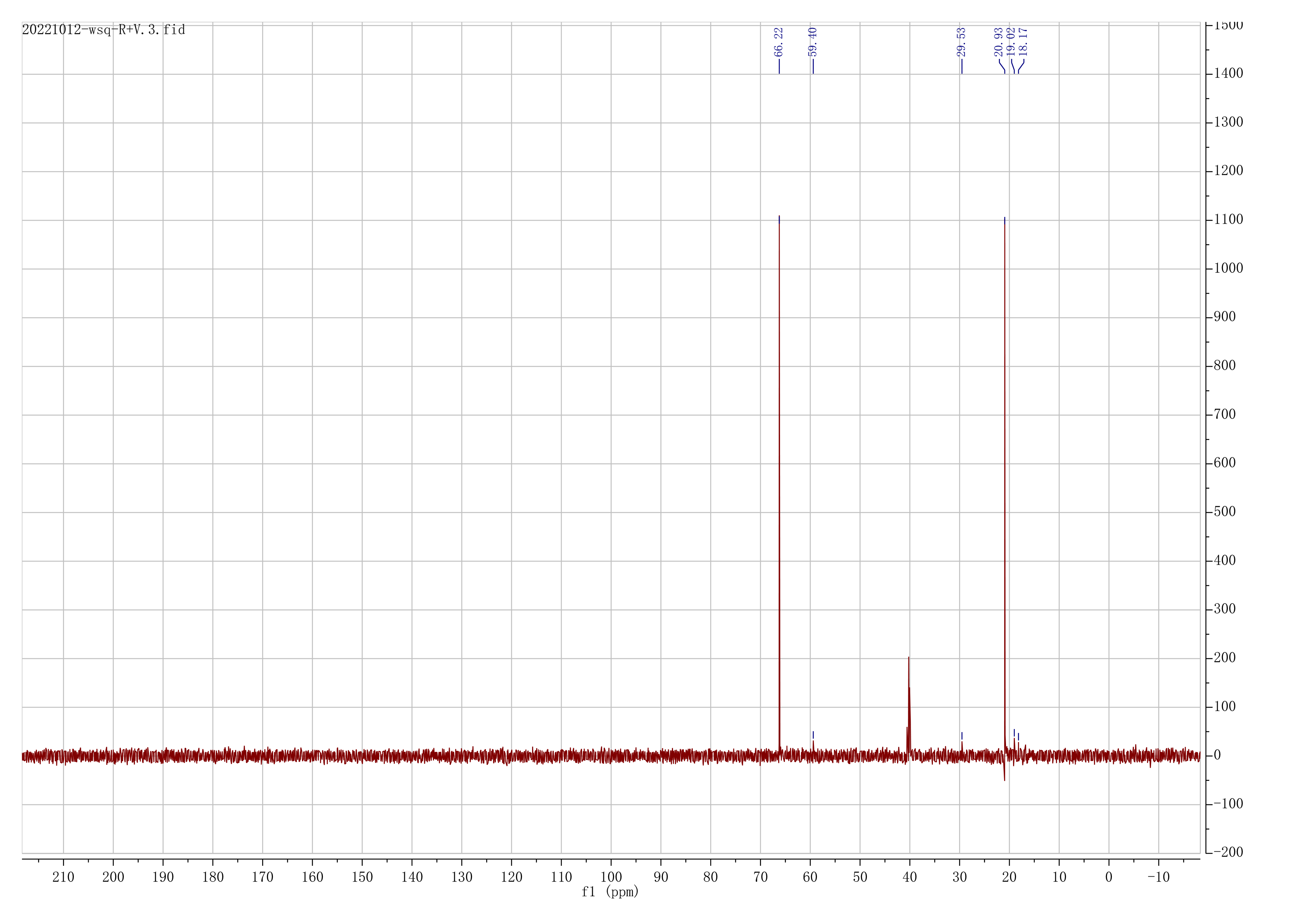

Supplement: Supplementary file 1 [file ijms-24-01986-s001.zip › Figure S22. DEPT spectrum of mixture of valine and LA in DMSO-d6.tif]

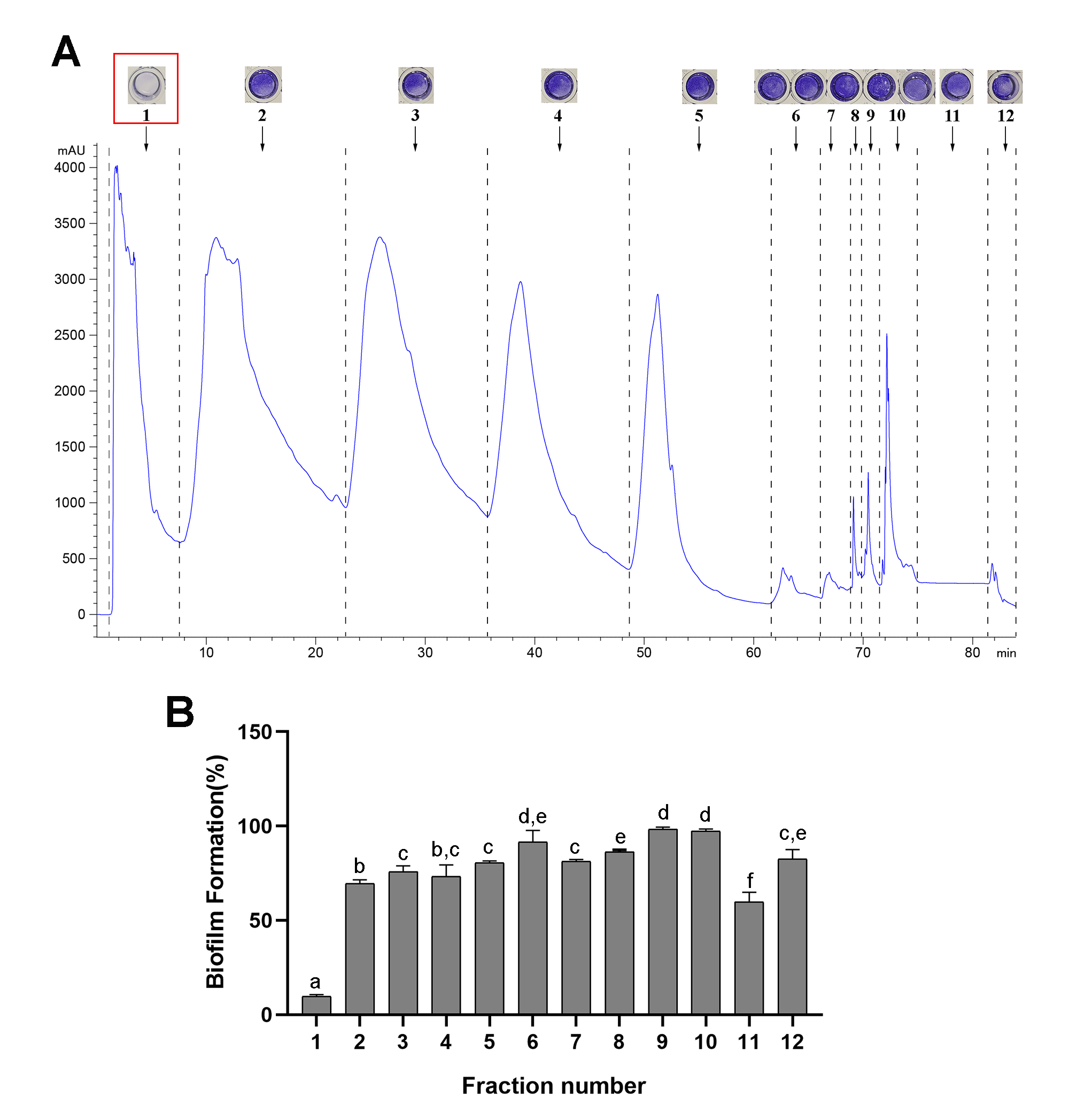

Supplement: Supplementary file 1 [file ijms-24-01986-s001.zip › Figure S3. Antibiofilm effect of fractions isolated by the ZORBAX SB-C18 column.tif]

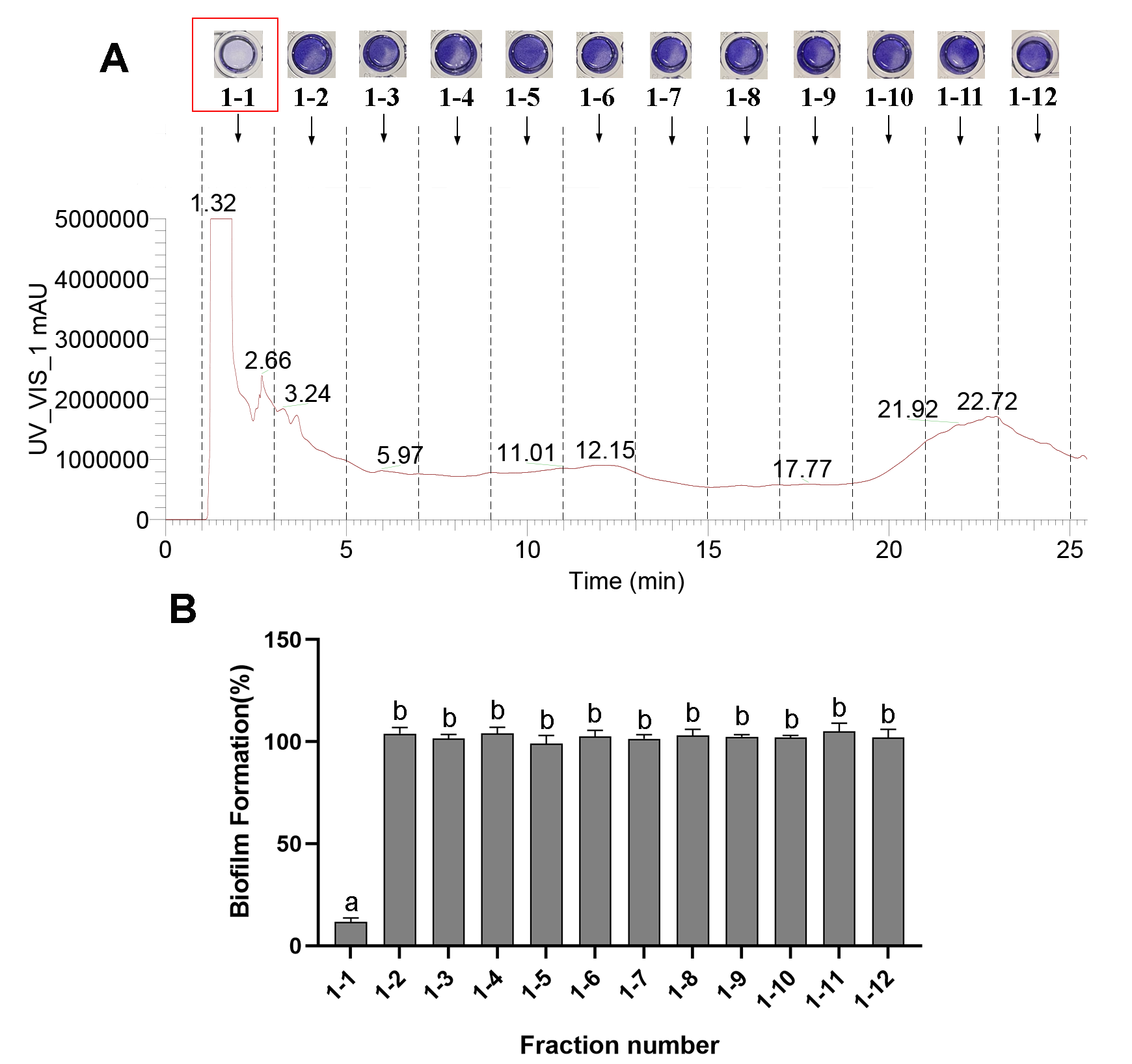

Supplement: Supplementary file 1 [file ijms-24-01986-s001.zip › Figure S4. Anti-biofilm effect of fractions isolated by the AccucoreTM HILIC column.tif]

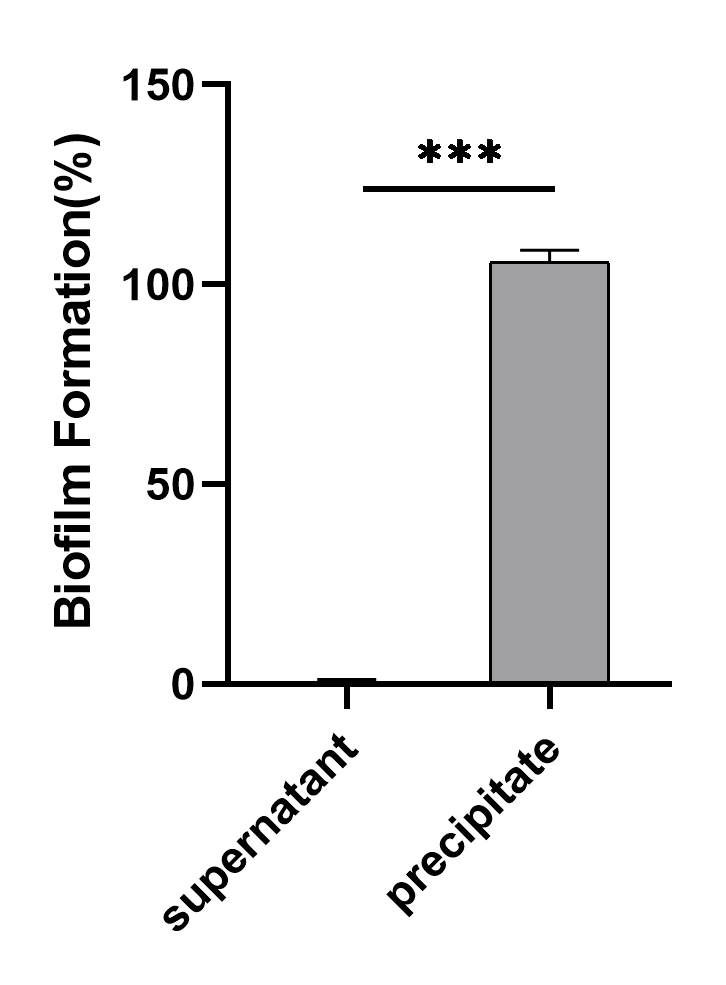

Supplement: Supplementary file 1 [file ijms-24-01986-s001.zip › Figure S5. Anti-biofilm activity of the supernatant and precipitate in the assay of ethanol precipitation.tif]

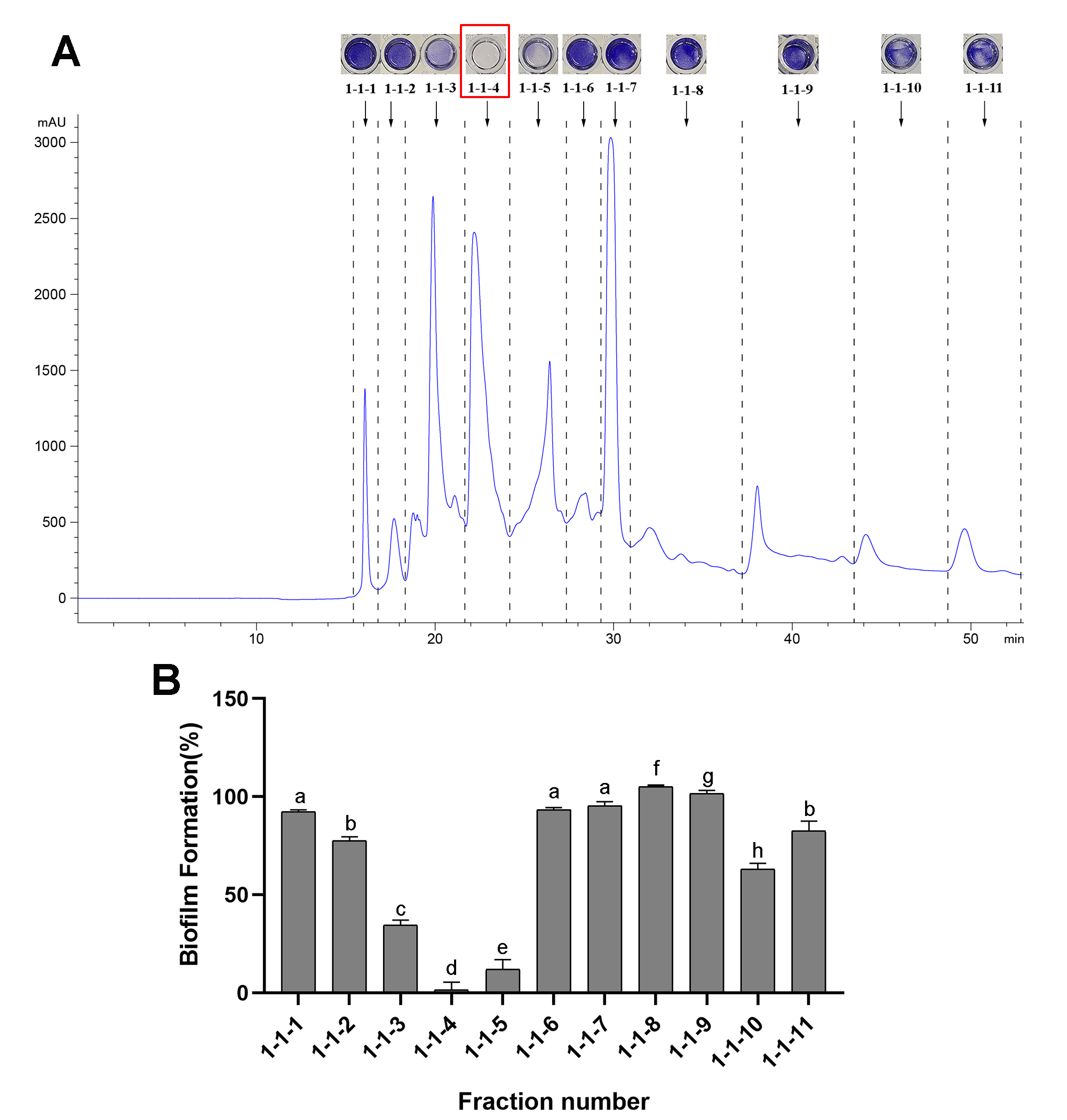

Supplement: Supplementary file 1 [file ijms-24-01986-s001.zip › Figure S6. Anti-biofilm effect of fractions isolated by the Eclipse XDB-C18 column.tif]

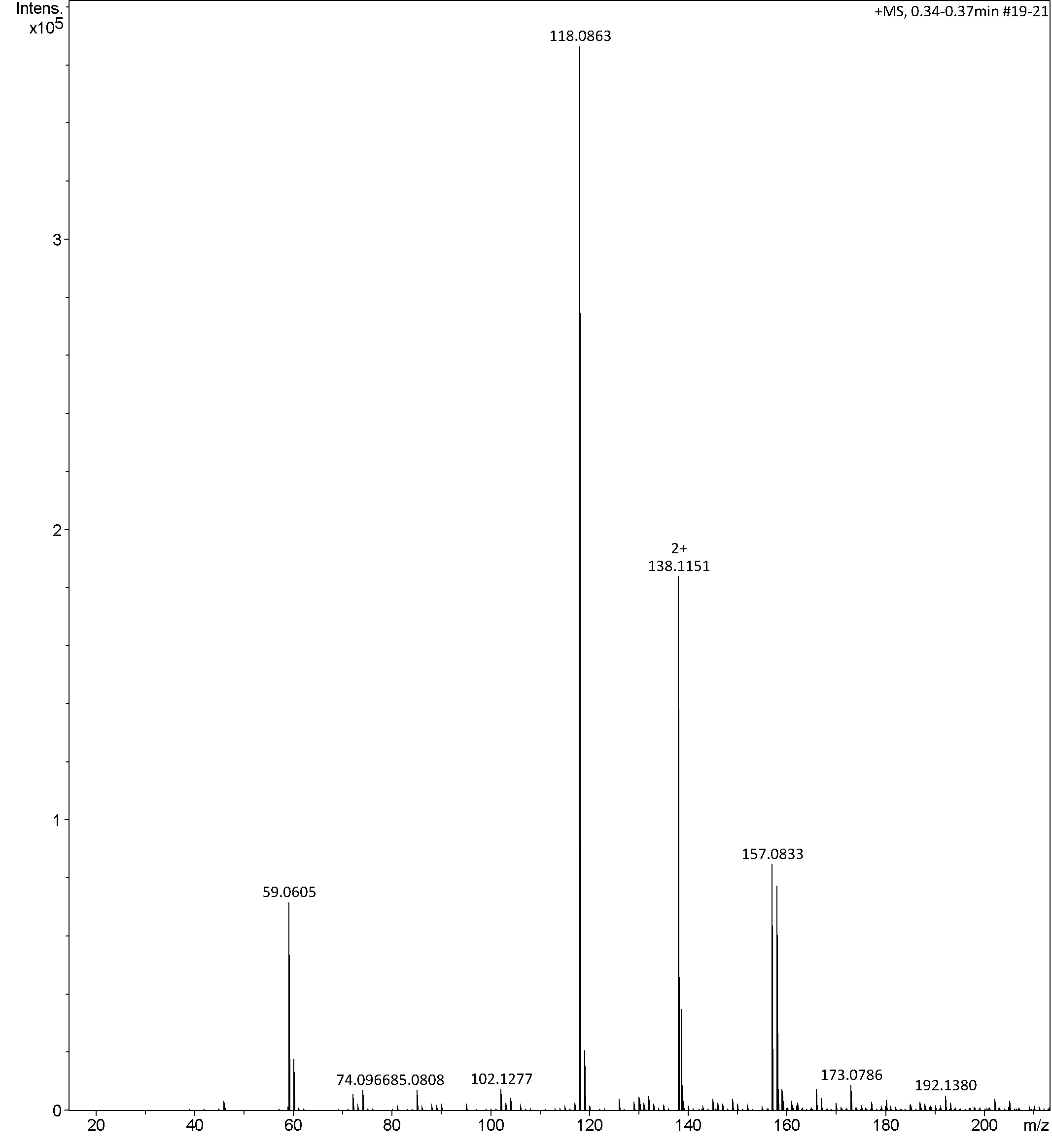

Supplement: Supplementary file 1 [file ijms-24-01986-s001.zip › Figure S7. Positive ESI-TOF spectrum of 1-1-4-3.tif]

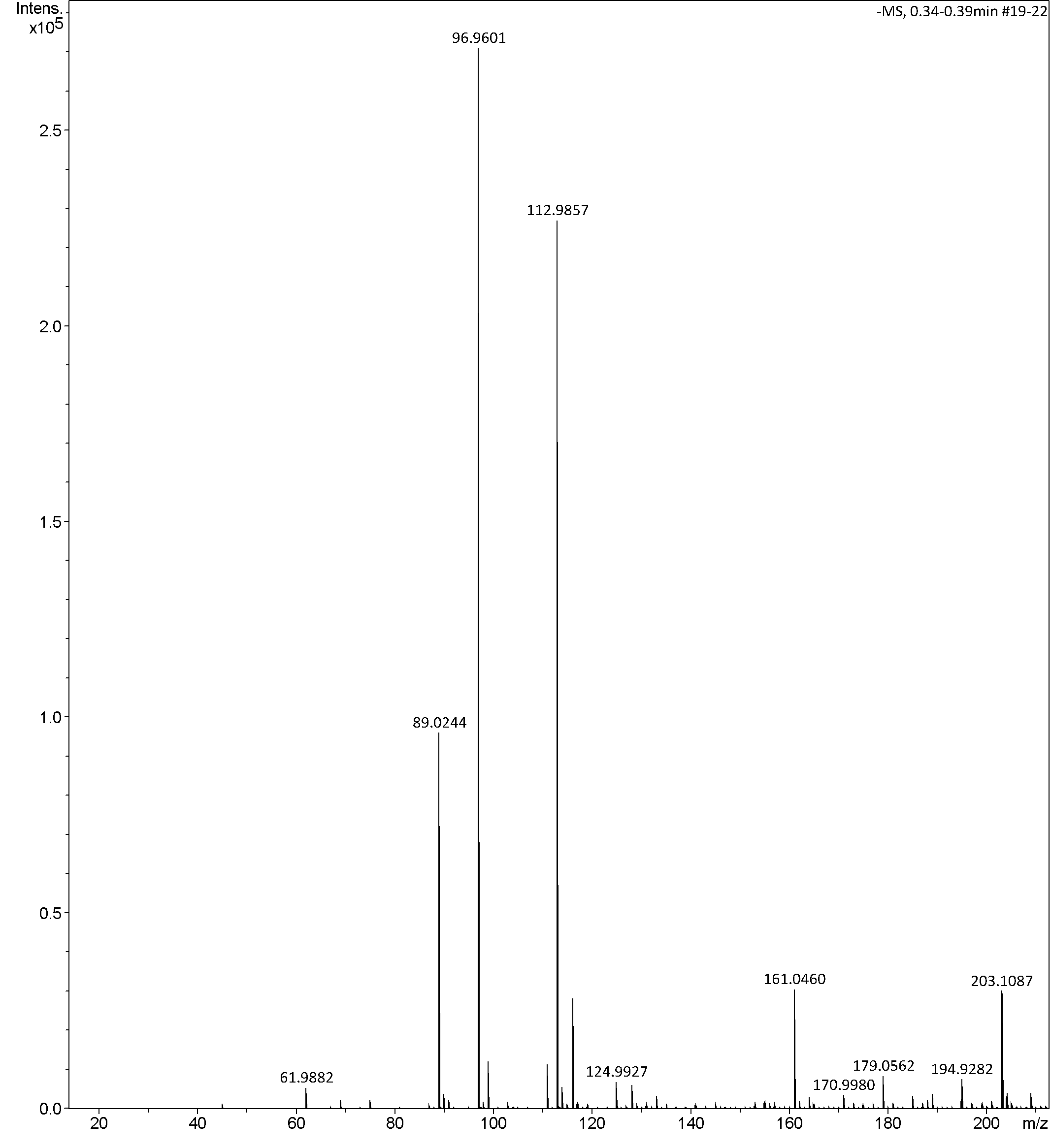

Supplement: Supplementary file 1 [file ijms-24-01986-s001.zip › Figure S8. Negative ESI-TOF spectrum of 1-1-4-3.tif]

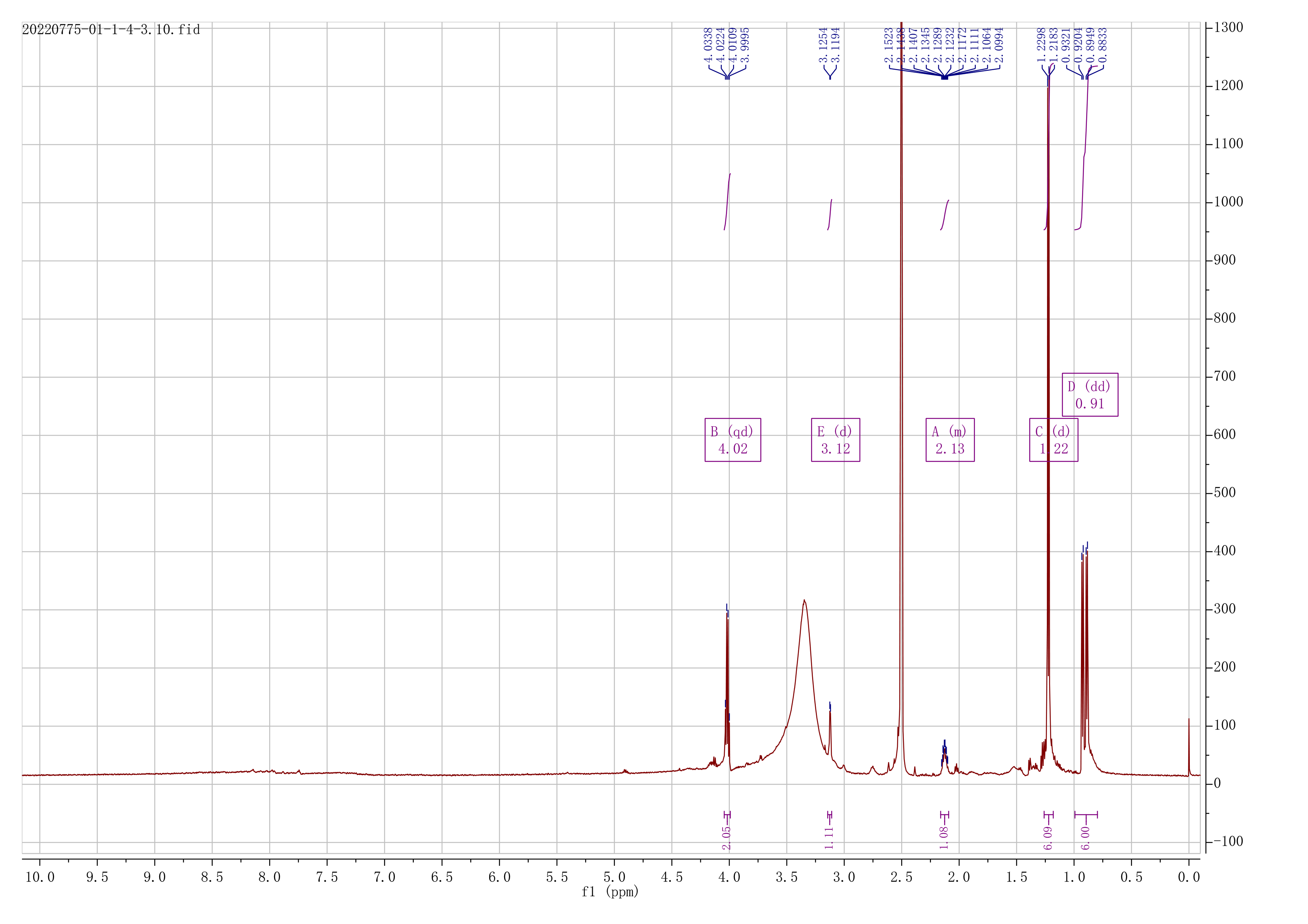

Supplement: Supplementary file 1 [file ijms-24-01986-s001.zip › Figure S9. 1H NMR spectrum of 1-1-4-3 in DMSO-d6.tif]
